# Development and Clinical Evaluation of a Wearable 12-Lead Electrocardiographic Platform with Automated ECG Analysis for Telemedicine Applications

**DOI:** 10.3390/s26175510

**Published:** 2026-08-30

**Authors:** Zhadyra Alimbayeva, Chingiz Alimbayev, Kassymbek Ozhikenov, Kairat Karibayev, Aiman Ozhikenova, Kymbat Khaidarova, Madiyar Daniyalov, Ussen Shylmyrza, Yerbolat Igembay, Akzhol Nurdanali

**Affiliations:** 1 Department of Robotics and Technical Means of Automation, Satbayev University, Almaty 050013, Kazakhstan; k.ozhikenov@satbayev.university (K.O.); a.ozhikenova@satbayev.university (A.O.); karibayev@snh.kz (K.K.); madiyar.daniyalov.131@gmail.com (M.D.); khaidarova.k@stud.satbayev.university (K.K.); u.shylmyrza@satbayev.university (U.S.); y.igembay@satbayev.university (Y.I.); a.nurdanali@satbayev.university (A.N.); 2 Department of Information Technology and Library Science, Kazakh National Women’s Teacher Training University, Almaty 050000, Kazakhstan; 3 Department of Disaster Medicine and Emergency Care Organization, Kazakh-Russian Medical University, Almaty 050013, Kazakhstan

**Keywords:** wearable electrocardiography, twelve-lead ECG, ESP32-S3, ADS1298, telemedicine, ST-segment analysis, ECG signal processing, cardiovascular monitoring

## Abstract

Wearable electrocardiographic technologies have become increasingly important for continuous cardiac monitoring; however, most existing portable systems are limited by the number of recorded leads or provide only basic signal acquisition without advanced automated analysis. This study presents a third-generation wearable twelve-lead electrocardiographic platform developed for multilead ECG acquisition and automated spatial ECG analysis. Compared with the previous generation, the hardware modification primarily consists of architectural consolidation: functions previously distributed across an STM32 microcontroller and separate wireless communication modules are integrated into a single ESP32-S3-based architecture, while the ECG acquisition principle, ten-electrode configuration, and sampling rate remain unchanged. The main methodological contribution of the present work is the software pipeline for lead-specific ST_80_ measurement and analysis of ST-segment deviations across anatomically contiguous leads. The system uses an ADS1298 analog front-end for synchronized multichannel ECG acquisition. The host software performs digital preprocessing, R-peak detection, ECG feature extraction, twelve-lead reconstruction, lead-specific ST_80_ measurement, contiguous-lead analysis, and generation of a preliminary computer-assisted ECG report. The developed platform was clinically evaluated using sequential recordings acquired with the proposed system and a reference clinical electrocardiograph. Quantitative comparison of automated PR, QRS, QT, and QTc measurements in 30 paired recordings demonstrated positive correlations with the reference BTL Flexi 12 ECG (r = 0.756–0.820, all *p* < 0.001), with mean absolute errors ranging from 2.53 ms for QRS duration to 10.40 ms for the QT interval. The system successfully recorded diagnostically interpretable twelve-lead ECGs in all participants and produced stable signal quality suitable for clinical assessment. The software automatically identified ECG waves and intervals, reconstructed twelve-lead recordings, evaluated ST-segment deviations across individual leads, and localized ischemia-related changes according to standard anatomical lead groups. Integration of signal acquisition, processing, visualization, and automated interpretation into a single telemedicine-oriented platform reduced hardware complexity while maintaining reliable multichannel ECG monitoring. The proposed wearable platform demonstrates the feasibility of combining compact embedded hardware with automated multilead ECG analysis for remote cardiovascular monitoring. The presented architecture provides a practical foundation for telemedicine applications and may support earlier recognition of clinically significant electrocardiographic abnormalities during ambulatory monitoring.

## 1. Introduction

In regions with low and middle levels of socio-economic development, a concerning increase in the incidence of cardiovascular diseases and related mortality has been observed. Epidemiological studies [1,2] report a significant growth in both new cases and deaths associated with cardiovascular diseases across South Asia, East Asia, Southeast Asia, Central Asia, and Central Latin America. Among cardiovascular conditions, acute coronary syndromes and myocardial infarction (MI) remain particularly critical pathologies [3], since early diagnosis and timely clinical intervention have a decisive impact on patient outcomes.

Electrocardiography (ECG) continues to be one of the most important diagnostic tools in contemporary cardiology [4]. Its wide availability, non-invasive nature, and diagnostic sensitivity explain why ECG is routinely used during the initial clinical evaluation of patients, regardless of whether cardiac symptoms are present. The standard twelve-lead ECG provides a spatial representation of cardiac electrical activity and enables clinicians to assess electrical propagation through different anatomical regions of the myocardium [5]. Such spatial information allows physicians to identify ischemic changes, conduction abnormalities, and other cardiac disorders with a relatively high degree of reliability. Despite the extensive use of clinical ECG systems in hospitals, developing wearable technologies that can provide comparable diagnostic information remains a complex engineering challenge.

Recent progress in microelectronics, embedded computing, wireless communication technologies, and digital signal processing has stimulated active development of wearable ECG monitoring systems [6]. These advances have enabled the creation of compact devices capable of continuously recording cardiac electrical activity outside clinical environments. Continuous monitoring is particularly valuable for patients with chronic cardiovascular diseases and individuals with an increased risk of cardiac events. Several types of wearable ECG devices have been introduced, including smartwatch-based sensors [7], adhesive ECG patches [8], chest belts [9], and textile-based monitoring systems with integrated electrodes [10]. Nevertheless, most currently available wearable ECG devices rely on recordings obtained from a single ECG lead or, in some cases, from two or three leads.

ECG devices based on a single lead provide only limited information about cardiac electrical activity [11]. Because the signal is recorded along a single projection vector, the spatial characteristics of cardiac depolarization and repolarization cannot be adequately evaluated. Such systems are suitable for monitoring heart rate, assessing heart rate variability, and detecting certain rhythm disturbances, including atrial fibrillation [12]. However, they do not provide sufficient information for identifying ischemic changes or diagnosing myocardial infarction. The electrocardiographic diagnosis of myocardial infarction is primarily based on the detection of ST-segment elevation or depression in anatomically contiguous leads together with the analysis of reciprocal changes, pathological Q waves, and T-wave inversion [13,14,15]. These diagnostic indicators appear across different spatial projections of the heart and therefore require simultaneous analysis of multiple ECG leads. When recordings are obtained from a single lead, the spatial distribution of these abnormalities cannot be evaluated, which makes such systems unsuitable for identifying ischemic patterns or determining the anatomical location of myocardial injury. Another limitation of wearable ECG systems that rely on a small number of leads is the inability to assess the electrical axis of the heart [16]. The conventional twelve-lead ECG allows clinicians to analyze cardiac electrical activity in both the frontal and horizontal planes. This information is essential for detecting axis deviation, bundle branch blocks, ventricular hypertrophy, and regional myocardial ischemia [17]. Without synchronized recordings from several leads, this type of spatial analysis cannot be performed. As a consequence, despite rapid technological progress in wearable monitoring devices, a noticeable gap still exists between consumer-oriented wearable ECG systems and clinical diagnostic electrocardiographs.

Multi-lead ECG recordings can be obtained using Holter monitoring systems. These systems are widely used for long-term cardiac observation. However, Holter monitoring is primarily designed for retrospective analysis of recorded signals. The evaluation of the collected data is usually performed after the monitoring period has been completed, which limits its usefulness in situations where rapid clinical assessment is required. In addition, traditional Holter systems are not intended for continuous real-time data transmission or remote medical evaluation in emergency conditions. Their wired electrode configuration may also affect patient comfort and can restrict monitoring during everyday activities [18]. These factors motivate the development of new wearable ECG technologies capable of providing multilead recordings together with real-time signal processing and wireless data transmission.

In our earlier work, a wearable ECG monitoring system based on recordings obtained from a single ECG lead was developed [19]. The system incorporated dedicated hardware architecture, signal filtering techniques, PQRST wave detection algorithms, and machine-learning approaches for anomaly identification. Although the device demonstrated reliable real-time ECG acquisition and rhythm-related analysis, its diagnostic capabilities were inherently limited by the use of a single ECG lead.

To overcome these limitations, the platform was subsequently extended to support synchronized acquisition of multiple ECG signals and reconstruction of a standard twelve-lead electrocardiogram [20]. The second generation of the platform enabled multilead ECG analysis and was evaluated for the assessment of electrocardiographic features associated with different glycemic states. However, its hardware architecture relied on a distributed processing approach in which an STM32 microcontroller performed data acquisition and processing, while separate ESP32 and HC-05 modules provided wireless communication. This configuration increased hardware complexity and required additional communication interfaces between individual modules.

**Figure 1:**
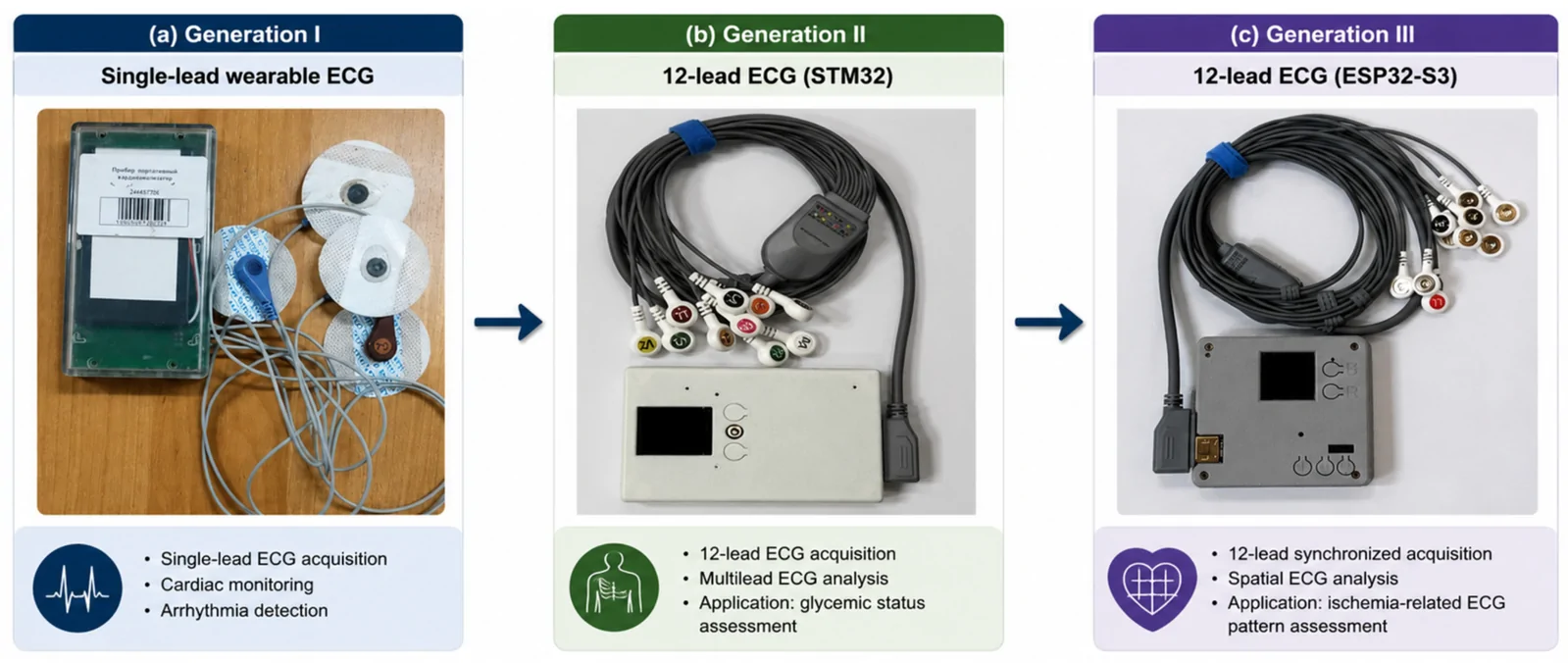
Evolution of the developed wearable ECG platform: (**a**) first-generation single-lead wearable ECG system for cardiac monitoring and arrhythmia detection [19]; (**b**) second-generation twelve-lead wearable ECG system based on a distributed STM32 architecture and developed for multilead ECG analysis [20]; and (**c**) the proposed third-generation twelve-lead wearable ECG system based on an integrated ESP32-S3 architecture for spatial ECG and ST-segment analysis.

The successive development of the platform reflects a transition from basic wearable ECG monitoring toward multilead acquisition and spatial ECG analysis. The three generations of the developed wearable ECG platform and their primary research applications are illustrated in Figure 1.

Building upon the multilead acquisition capability of the second-generation platform, the present work focuses on further architectural optimization and the extension of the system toward spatial ECG analysis. Assessment of ischemia-related electrocardiographic abnormalities requires reliable acquisition of synchronized ECG leads, sufficient computational resources for processing multilead data streams, and analysis of ST-segment deviations across anatomically contiguous leads. These requirements motivated the development of an integrated hardware architecture capable of supporting multilead acquisition and subsequent analysis of spatial ECG features.

In the present study, the hardware architecture of the previously developed multilead ECG platform [20] was revised by consolidating functions that had been distributed across separate processing and communication modules into a single ESP32-S3-based architecture. This modification represents an engineering optimization rather than a change in the fundamental ECG acquisition approach. The ten-electrode configuration, synchronized multichannel acquisition, sampling frequency, and subsequent PC-based reconstruction of the standard twelve-lead ECG were retained from the previous generation [20]. The consolidated architecture was introduced to reduce hardware complexity, the number of intermodule connections, and PCB dimensions.

The main methodological contribution of the present study is the extension of the software framework toward spatial analysis of the twelve-lead ECG. The implemented processing pipeline performs lead-specific ST_80_ measurement and evaluates ST-segment deviations across anatomically contiguous lead groups corresponding to anterior, inferior, and lateral myocardial regions. These measurements are integrated with ECG interval and waveform analysis to provide preliminary computer-assisted assessment of regional ischemia-related ECG patterns.

Accordingly, the objective of this study was to evaluate the consolidated third-generation wearable ECG platform and the associated multilead analysis software. Technical and clinical evaluation included comparison of ECG recordings with the BTL FLEXI clinical twelve-lead electrocardiograph, while the software component was assessed with respect to automated ECG interval measurement, lead-specific ST_80_ assessment, and contiguous-lead analysis. The automated interpretation is intended as a decision-support function and does not replace clinical diagnosis.

## 2. Materials and Methods

### 2.1. Hardware Architecture Evolution

**Figure 2:**
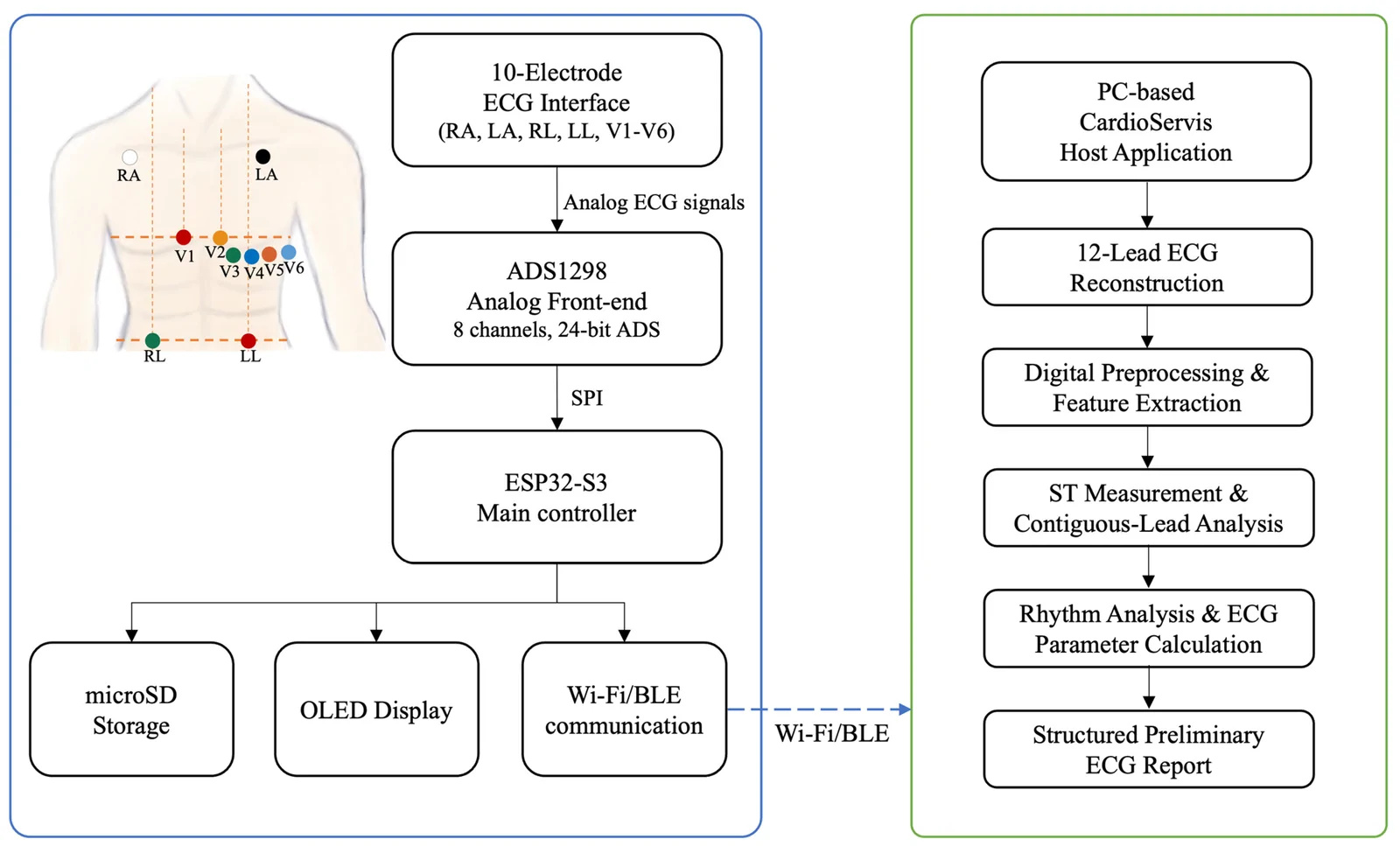
Functional architecture and data flow of the proposed wearable twelve-lead ECG platform.

The system architecture includes a ten-electrode acquisition interface, an ADS1298 analog front-end, an ESP32-S3 main controller with integrated wireless connectivity, local data storage, power-management circuitry, and a PC-based host application. The ESP32-S3 controls synchronized ECG acquisition, data buffering, local storage, display management, and wireless transmission, whereas twelve-lead ECG reconstruction and subsequent automated signal analysis, including ST-segment assessment, are performed by the host application. A functional overview of the proposed system is shown in Figure 2. To improve the readability of the manuscript, only the functional architecture is presented in the main text, whereas the complete component-level electrical schematic of the device is provided in the Supplementary Materials (Figure S1).

#### Evolution of the Wearable ECG Platform

The proposed system represents the third generation of the wearable ECG platform developed by the authors. Its principal hardware modification is the transition from the distributed architecture of the preceding generation, in which processing and wireless communication functions were implemented using separate modules, to a consolidated ESP32-S3-based architecture. The ECG acquisition principle itself was not changed: the system retains the ten-electrode configuration, ADS1298 analog front-end, synchronized multichannel acquisition, and subsequent reconstruction of the standard twelve-lead ECG. The purpose of the hardware redesign was therefore to reduce architectural complexity and the number of intermodule connections while maintaining the multilead acquisition capability required by the software analysis pipeline. Table 1 summarizes the principal hardware and functional characteristics of the three platform generations. Table 1 summarizes the architectural evolution and the principal hardware and functional characteristics of the three platform generations.

**Figure 3:**
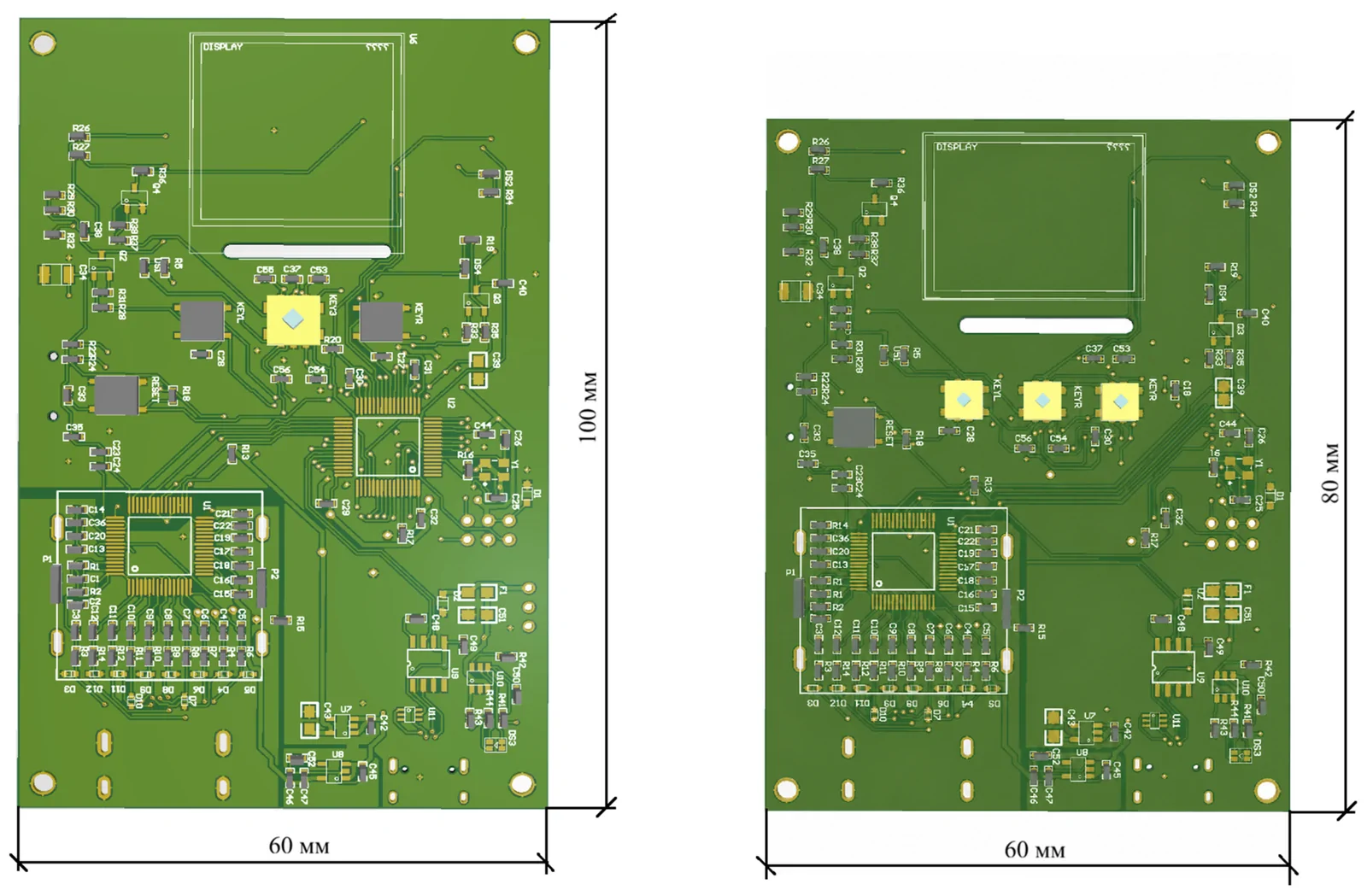
PCB layouts of the second- and third-generation wearable 12-lead ECG systems. The third-generation board integrates signal processing and wireless communication into a single ESP32-S3 system-on-chip, resulting in a more compact hardware implementation.

The comparison presented in Table 1 shows that the proposed platform preserves the multilead acquisition principle of the previous generation while consolidating processing and communication functions within a single ESP32-S3-based architecture. Compared with the STM32-based configuration used previously, the ESP32-S3 integrates Wi-Fi and Bluetooth Low Energy connectivity and provides the processing and memory resources required for device control and synchronized multichannel data handling. This consolidation reduced the number of separate hardware modules and associated interconnections. The PCB layouts of the second- and third-generation platforms are compared in Figure 3.

In the proposed system, the STM32 microcontroller and external communication modules were replaced by a single ESP32-S3 system-on-chip. The ESP32-S3 integrates signal acquisition control, communication with the ADS1298 analog front-end, local data storage, display management, wireless transmission, and embedded signal-processing functions within a single controller. Consolidation of these functions within a single controller significantly simplified the hardware architecture and reduced the number of intermodule connections.

An additional advantage of the proposed architecture is the reduction in the overall PCB area. Although the board width remained unchanged at 60 mm, the PCB height was reduced from 100 mm to 80 mm. This dimensional reduction corresponds to a 20% decrease in the total PCB area, directly contributing to a more compact wearable device.

The greater processing and memory resources of the ESP32-S3 facilitate synchronized multichannel data handling and device-level operations within the consolidated architecture. In the present implementation, however, twelve-lead reconstruction and the principal ECG analysis procedures, including ST_80_ measurement and contiguous-lead analysis, are performed by the PC-based host software. Thus, the role of the ESP32-S3 in the current platform is primarily to simplify the embedded hardware architecture while supporting reliable acquisition, buffering, storage, and transmission of multichannel ECG data.

The ESP32-S3 integrates Wi-Fi and Bluetooth Low Energy communication interfaces within the same chip. As a result, external communication modules and dedicated UART-based interconnections are no longer required. The controller also provides native USB OTG functionality, enabling direct connection of the device to a personal computer for data transfer, firmware updates, and system configuration.

Through architectural integration and hardware optimization, the proposed platform achieves lower complexity, reduced power consumption, and improved computational performance while maintaining the capability of synchronized acquisition and reconstruction of a standard twelve-lead electrocardiogram. These improvements provide the technical foundation for real-time analysis of spatial ECG features associated with myocardial ischemia and other clinically significant cardiovascular abnormalities.

### 2.2. Software Architecture

**Figure 4:**
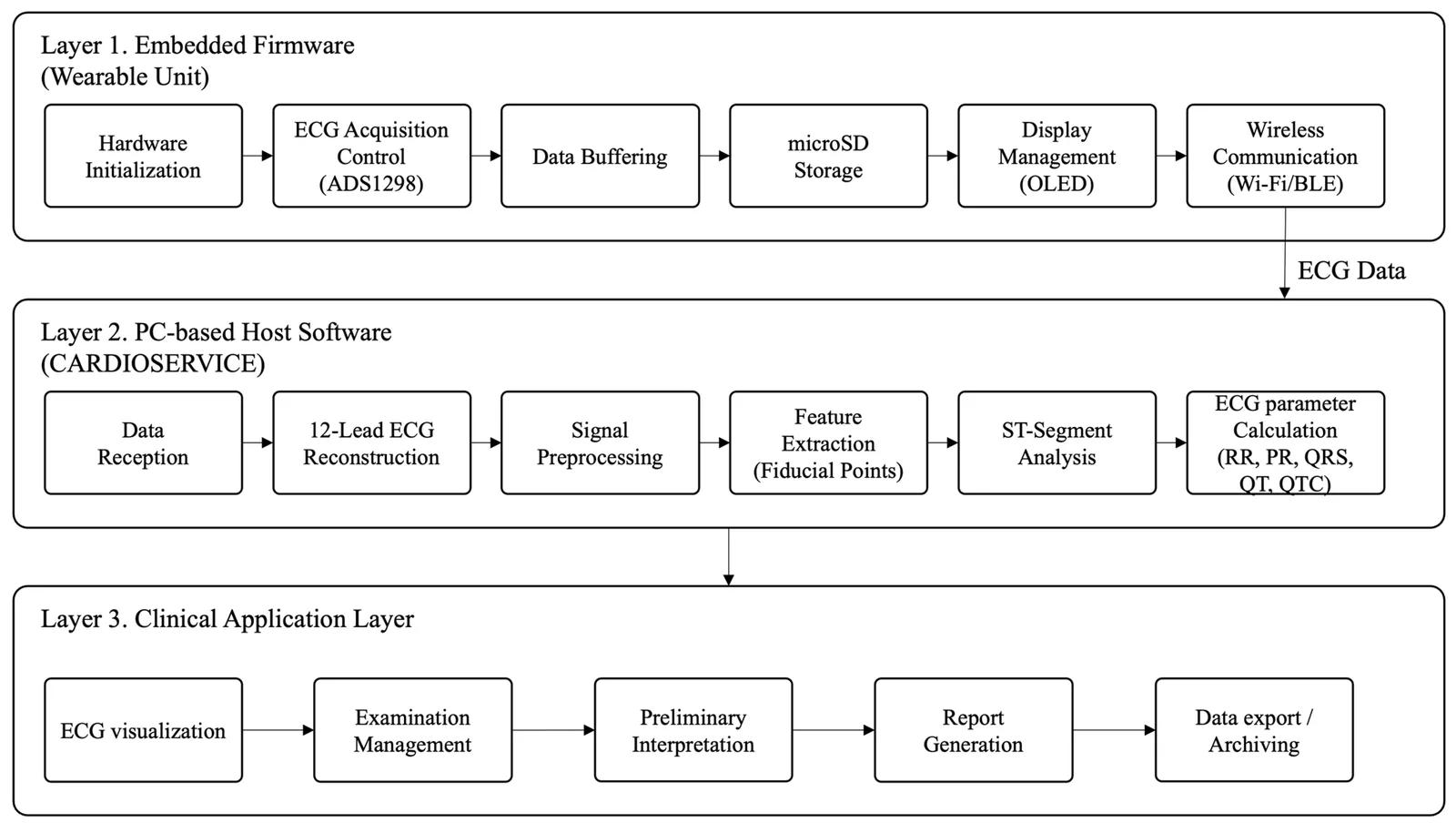
Three-layer software architecture of the proposed ECG platform, comprising embedded firmware, the PC-based CardioService host software, and the clinical application layer.

The software architecture of the proposed wearable electrocardiographic system was designed to provide reliable real-time acquisition, processing, visualization, and management of multichannel ECG signals while maintaining low computational overhead on the embedded platform. The architecture consists of three functional layers: (i) embedded firmware responsible for real-time data acquisition and device control, (ii) host software performing ECG reconstruction and signal analysis, and (iii) the clinical application layer providing visualization, automatic interpretation, and report generation. The overall software architecture is presented in Figure 4.

As illustrated in Figure 4, the embedded firmware performs synchronized ECG acquisition, local buffering, device control, and wireless communication. The host application reconstructs the standard twelve-lead ECG, executes automatic signal processing algorithms, and manages examination records. Computationally intensive processing is therefore shifted from the wearable device to the host software, improving system efficiency while preserving real-time acquisition performance.

#### 2.2.1. Embedded Firmware

The embedded software was developed using the ESP-IDF framework (version 6.0) for the ESP32-S3 microcontroller. During system initialization, the firmware configures all hardware peripherals, including the ADS1298 analog front-end, SPI, OLED display, microSD storage, USB interface, wireless communication modules, and user control buttons.

Following initialization, ECG acquisition is performed using hardware interrupts generated by the ADS1298 Data Ready (DRDY) signal. Each interrupt indicates the availability of a new synchronized sample from all acquisition channels. The firmware immediately retrieves the digital samples through the SPI, stores them in an internal memory buffer, and forwards them to the subsequent processing and communication modules. This interrupt-driven architecture guarantees deterministic sampling intervals and minimizes timing jitter during continuous multichannel acquisition.

To improve system reliability, acquired ECG samples are simultaneously stored on the onboard microSD card and transmitted wirelessly to the host application. Local storage prevents data loss during temporary communication interruptions, whereas wireless transmission enables real-time visualization and remote analysis of ECG recordings.

The firmware also manages the OLED display, continuously updating device status information including electrode connection state, acquisition progress, wireless connectivity, battery level, and recording status. User interaction is implemented through dedicated hardware buttons for power management, ECG recording control, and event marking during long-term monitoring.

#### 2.2.2. Wireless Communication and Data Management

The ESP32-S3 integrates both Wi-Fi and Bluetooth Low Energy communication interfaces within a single system-on-chip, eliminating the need for dedicated external communication modules used in previous generations of the wearable platform. This integration considerably simplifies the hardware architecture while improving communication reliability and reducing overall power consumption.

Wireless communication enables continuous transmission of ECG recordings to the host application for real-time visualization and storage. In addition to live monitoring, the communication subsystem supports synchronization of examination records, firmware maintenance, and rapid export of recorded ECG data for subsequent clinical evaluation.

To ensure uninterrupted operation during unstable network conditions, all acquired ECG recordings are simultaneously archived on the onboard microSD memory card. Consequently, complete examination records remain available even if wireless connectivity is temporarily interrupted, thereby improving system robustness for ambulatory monitoring.

#### 2.2.3. Host Application

In the present implementation, the host application was executed on a personal computer (PC). No mobile-phone or server-side processing was used during the experiments reported in this study. The wearable unit transmitted the acquired ECG data to the PC, where the CardioService software performed twelve-lead ECG reconstruction and subsequent automated signal analysis. Accordingly, the current prototype employs a two-level processing architecture: the ESP32-S3-based wearable unit is responsible for acquisition-related and communication functions, whereas computational ECG reconstruction and interpretation are performed by the PC-based host application.

A dedicated software application was developed to support visualization, management, analysis, and storage of multichannel ECG recordings. The application serves as the primary interface between the wearable ECG device and clinical users, providing synchronized visualization of all reconstructed twelve ECG leads together with patient examination records.

The host application performs automatic reconstruction of the standard twelve-lead ECG from the synchronized electrode recordings received from the wearable device. Reconstructed signals are displayed simultaneously, allowing comprehensive assessment of cardiac electrical activity across both frontal and horizontal anatomical planes.

In addition to real-time visualization, the application provides centralized management of examination records, allowing efficient retrieval, review, long-term storage, and export of ECG recordings for both clinical evaluation and research purposes.

Automatic ECG interpretation is incorporated as a decision-support tool rather than a replacement for clinical judgment. Following signal acquisition, the application performs digital signal preprocessing, cardiac interval estimation, rhythm assessment, ST-segment evaluation, and generation of a preliminary automated report. Therefore, the proposed system should be considered as a clinical decision-support tool rather than a fully autonomous diagnostic system.

#### 2.2.4. Software Workflow

**Figure 5:**
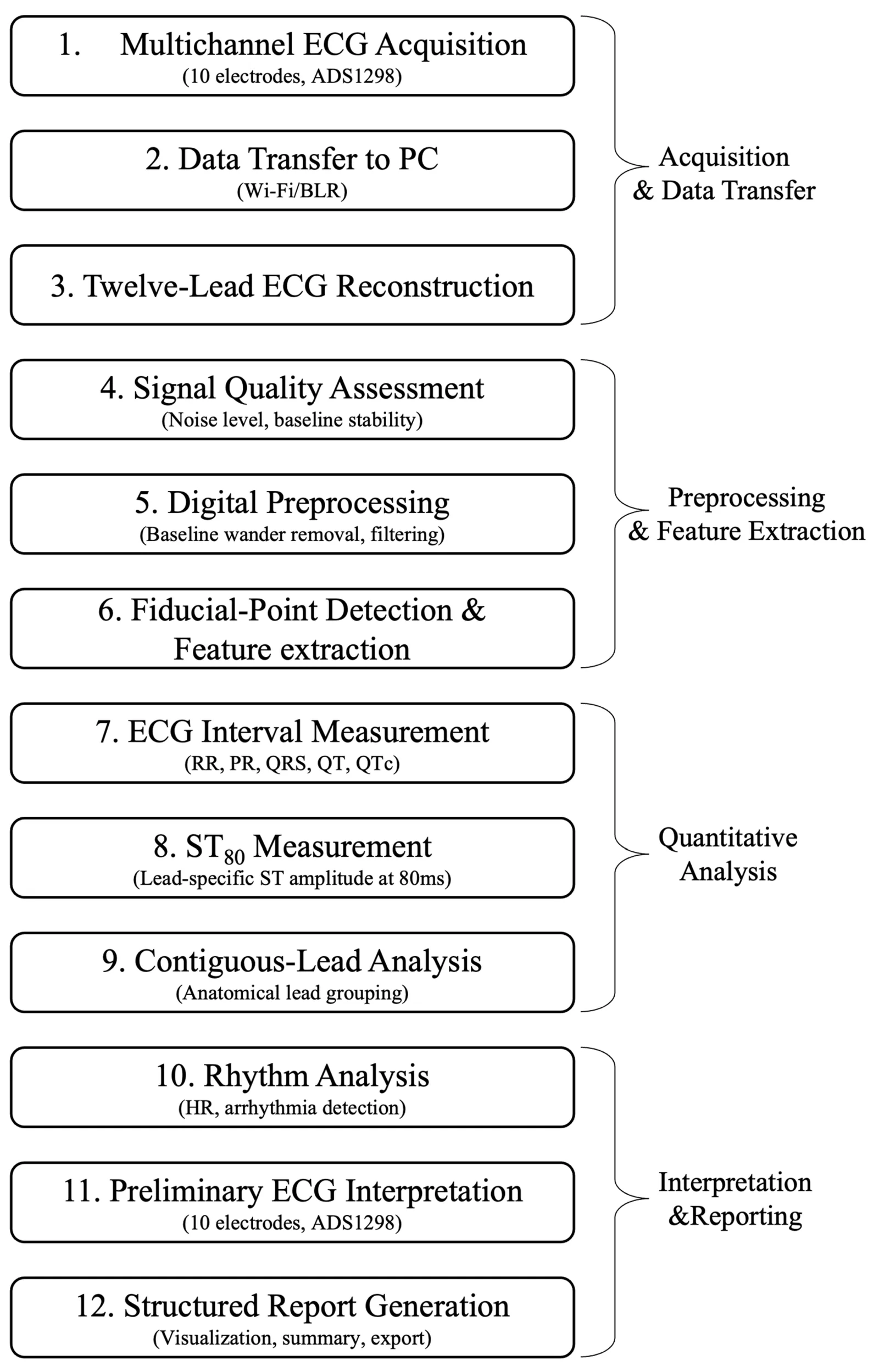
Signal-processing workflow of the proposed platform, from multichannel ECG acquisition and twelve-lead reconstruction to automated feature extraction, ST_80_ assessment, contiguous-lead analysis, and structured report generation.

The complete software workflow is summarized in Figure 5. After initialization of the embedded system, synchronized ECG signals are continuously acquired from the ADS1298 analog front-end. The acquired samples are buffered, stored locally, and transmitted wirelessly to the host application.

The PC-based host software subsequently reconstructs the twelve standard ECG leads, performs digital signal preprocessing and signal-quality assessment, executes automatic feature extraction and fiducial-point detection, calculates clinically relevant ECG intervals, performs lead-specific ST_80_ measurement and contiguous-lead analysis, and generates the structured preliminary report. The processed data are visualized in real time and archived together with examination metadata for future clinical review. Automatic analysis results are presented as preliminary diagnostic assistance and require confirmation by a physician before clinical use.

The software workflow was designed as a sequential processing pipeline in which each processing stage receives the output of the previous module. Such a modular architecture facilitates future implementation of additional signal processing algorithms without modification of the acquisition subsystem. Furthermore, separation of individual processing stages improves software maintainability and computational efficiency.

A detailed description of each signal processing stage is presented in Section 2.3.

The main software modules and their corresponding functions are summarized in Table 2.

### 2.3. Signal Processing Methodology

The signal processing algorithms implemented in the proposed wearable ECG platform were designed to extract clinically relevant electrocardiographic parameters from synchronized multichannel recordings while preserving waveform morphology. As illustrated in Figure 5, the proposed processing framework consists of consecutive stages including signal acquisition, quality assessment, digital preprocessing, feature extraction, twelve-lead ECG reconstruction, ST-segment evaluation, rhythm analysis, and automatic interpretation. Each processing stage is described in detail in the following subsections.

The signal processing workflow presented in Figure 3 is described in detail in the following subsections.

#### 2.3.1. Signal Acquisition and Quality Assessment

Accurate extraction of clinically relevant electrocardiographic parameters requires reliable acquisition of high-quality ECG signals. However, wearable ECG recordings are frequently affected by baseline fluctuations, power-line interference, motion artifacts, muscle activity, and variations in electrode-skin contact. These factors may substantially degrade signal quality and adversely influence the accuracy of subsequent feature extraction and automated ECG interpretation. Therefore, synchronized multichannel acquisition and signal quality assessment were incorporated as the initial stages of the proposed signal processing pipeline.

The developed wearable ECG system acquires synchronized bioelectrical signals from ten electrodes using the ADS1298 analog front-end, allowing reconstruction of the standard twelve-lead electrocardiogram. The analog signals are simultaneously digitized by the ADS1298 and transferred to the ESP32-S3 microcontroller via the Serial Peripheral Interface (SPI). The embedded firmware continuously monitors the Data Ready (DRDY) signal generated by the ADS1298, ensuring deterministic acquisition of synchronized ECG samples from all channels. The acquired data are temporarily buffered in memory before being simultaneously stored on the onboard microSD card and transmitted wirelessly to the host application for further processing.

The sampling frequency determines the temporal resolution of the acquired ECG signal and must satisfy the Nyquist sampling criterion

$$f_{s}\geq {2f}_{max}$$

where fs is the sampling frequency and fmax represents the highest frequency component of the ECG signal. In the proposed system, a sampling frequency of 500 Hz was selected to ensure accurate representation of the P wave, QRS complex, ST segment, and T wave while maintaining sufficient temporal resolution for subsequent signal processing.

The corresponding sampling interval is given by

$$T_{s}=\frac{1}{f_{s}}$$

where *T_s_* is the sampling interval and *f_s_* is the sampling frequency. For *f_s_* = 500 Hz, the sampling interval is *T_s_* = 2 ms.

$${SNR}_{HF}=\frac{\sigma \left(s\right)}{\sigma \left(HF>40\;\mathrm{Hz}\left(s\right)\right)}$$

where s denotes the acquired ECG signal, σ(⋅) represents the standard deviation, and *HF* > 40 Hz(s) corresponds to the high-frequency component of the signal obtained after high-pass filtering above 40 Hz. This metric estimates the relative contribution of useful ECG information with respect to high-frequency noise while preserving diagnostically important waveform morphology.

Based on the calculated SNR, each ECG channel is automatically assigned to one of five predefined quality categories. Recordings with insufficient signal quality are excluded from automatic ECG interpretation until acceptable signal quality is restored. In addition, channels exhibiting an almost flat signal with a standard deviation below 1 μ_V_ are automatically classified as no-contact, indicating inadequate electrode-skin contact. This preliminary quality assessment improves the robustness of subsequent preprocessing, feature extraction, and ST-segment analysis by preventing unreliable measurements derived from low-quality recordings.

Table 3 summarizes the signal quality classification criteria implemented in the proposed software platform.

#### 2.3.2. ECG Signal Preprocessing

The raw ECG signals acquired from wearable devices are commonly contaminated by various types of noise, including baseline wander caused by respiration, power-line interference, high-frequency electronic noise, and motion artifacts. These disturbances may significantly affect the accuracy of subsequent ECG feature extraction and diagnostic analysis. Therefore, an effective preprocessing stage is essential to suppress unwanted noise while preserving the morphology of clinically important waveform components such as the P wave, QRS complex, ST segment, and T wave.

To minimize signal distortion, the proposed system employs a sequential digital filtering pipeline consisting of a high-pass Butterworth filter, a notch filter, and a low-pass Butterworth filter. Zero-phase filtering is implemented using forward and reverse filtering (filtfilt), thereby eliminating phase distortion and preserving the temporal characteristics of the ECG waveform.

The preprocessing pipeline consists of the following stages: (1) high-pass filtering (0.5 Hz) to suppress baseline wander caused by respiration and slow body movements; (2) Notch filtering (50 Hz) to eliminate power-line interference; (3) low-pass filtering (40 Hz) to remove high-frequency noise and muscle artifacts while preserving diagnostically relevant ECG components.

The normalized cutoff frequency of each digital filter is calculated as

$$f_{n}=\frac{f_{c}}{f_{s}/2}$$

where fc is the cutoff frequency and *f_s_* is the sampling frequency.

The characteristics of the implemented digital filters are summarized in Table 4.

**Figure 6:**
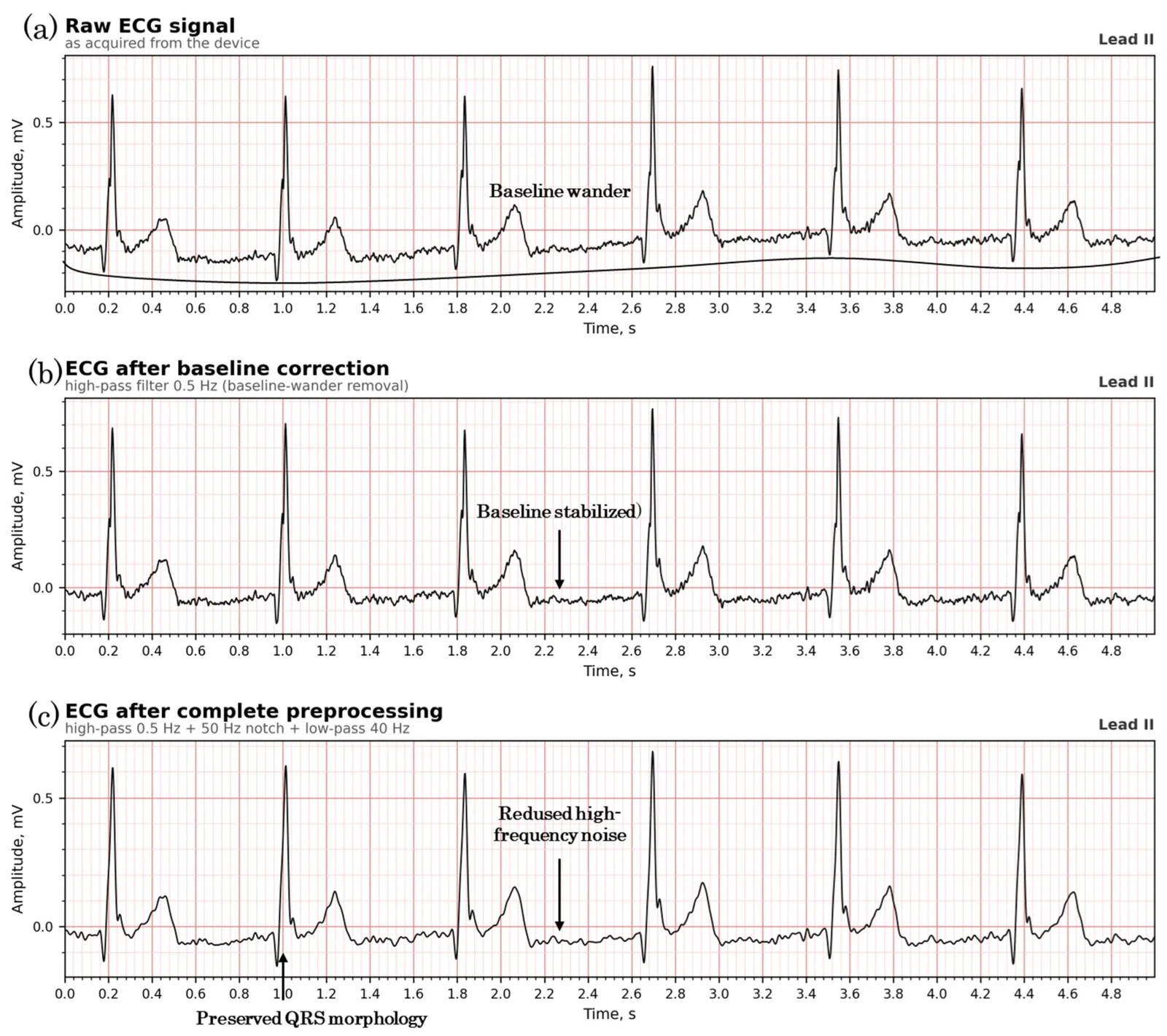
Effect of the proposed ECG preprocessing pipeline. (**a**) Raw ECG acquired from the wearable device. (**b**) ECG after baseline-wander removal using a 0.5 Hz high-pass filter. (**c**) ECG after complete preprocessing, including high-pass filtering, 50 Hz notch filtering, and 40 Hz low-pass filtering, demonstrating baseline stabilization, suppression of high-frequency noise, and preservation of ECG morphology.

Following digital filtering, the preprocessed ECG signals are forwarded to the feature extraction stage. The filtering procedure substantially improves signal quality while maintaining the morphology of diagnostically significant ECG components. Preservation of waveform morphology is particularly important for accurate measurement of cardiac intervals and reliable assessment of ST-segment deviations, which constitute the primary diagnostic targets of the proposed wearable ECG platform. The effect of the sequential preprocessing stages on a representative ECG recording is illustrated in Figure 6.

#### 2.3.3. R-Peak Detection and Beat Segmentation

Reliable detection of R-peaks is a fundamental step in automated ECG analysis because it provides temporal reference points for cardiac cycle segmentation and subsequent measurement of clinically relevant electrocardiographic parameters. Accurate localization of R-peaks directly influences the estimation of heart rate, RR intervals, PR interval, QRS duration, QT interval, and ST-segment position. Consequently, robust R-peak detection is essential for reliable ECG interpretation.

In the proposed system, R-peak detection is performed using Lead II, which generally provides the highest signal amplitude and the most distinct QRS morphology among the standard ECG leads. The processing pipeline primarily employs the NeuroKit2 library (version 0.2.13) for ECG delineation. When this library is unavailable, a custom implementation based on the Pan–Tompkins algorithm is automatically executed to ensure uninterrupted signal analysis.

The implemented detection algorithm consists of the following sequential stages:

Band-pass filtering (5–15 Hz) to enhance the QRS complex while suppressing baseline drift and high-frequency noise.

Signal differentiation to emphasize rapid changes associated with ventricular depolarization.

Squaring of the differentiated signal to amplify high-frequency components and eliminate polarity dependence.

Moving-window integration using a window length of approximately 120 ms to estimate local signal energy.

Adaptive thresholding for preliminary identification of candidate R-peaks.

Peak refinement by locating the maximum absolute amplitude of the original ECG signal within a ±50 ms search window.

The adaptive detection threshold is calculated as

$$\theta =\mu_{I}+0.5\sigma_{I}$$

where $\mu_{I}$ and $\sigma_{I}$ denote the mean value and standard deviation of the integrated signal, respectively.

Following R-peak detection, individual cardiac cycles are segmented according to consecutive RR intervals. The duration of each RR interval is determined as

$${RR}_{i}=\frac{R_{i+1}-R_{i}}{f_{s}}$$

where *R_i_* and *R_i+_*_1_ are the sample indices of two consecutive R-peaks and *f_s_* is the sampling frequency. The mean RR interval is subsequently calculated across the accepted cardiac cycles, and heart rate is estimated as

$$HR=\frac{60}{\bar{RR}}$$

where $\bar{RR}$ is expressed in seconds and HR in beats per minute.

The detected R-peaks are further utilized as reference landmarks for beat segmentation. Each cardiac cycle is extracted within predefined temporal windows surrounding the detected R-wave, enabling accurate localization of the P wave, QRS complex, ST segment, and T wave during subsequent feature extraction. This segmentation strategy provides consistent temporal alignment of individual cardiac beats and improves the robustness of interval measurements and morphological analysis across all reconstructed ECG leads.

To minimize false detections, physiologically implausible RR intervals are discarded during post-processing. Only RR intervals within the range of 0.35–1.80 s are retained for subsequent analysis, thereby reducing the influence of motion artifacts and spurious detections on heart rate estimation and feature extraction.

A dedicated PC-based host application, CardioService, was developed by the authors specifically for the proposed ECG platform to support ECG visualization, twelve-lead reconstruction, automated signal analysis, examination management, and data storage.

#### 2.3.4. 12-Lead ECG Reconstruction

The standard twelve-lead electrocardiogram represents cardiac electrical activity in six frontal-plane leads and six horizontal-plane leads. Although twelve signals are displayed, they are obtained using ten physical electrodes: four limb electrodes—right arm (RA), left arm (LA), right leg (RL), and left leg (LL)—and six precordial electrodes positioned at V1–V6. The RL electrode serves as the reference or driven-right-leg electrode and is not used directly in lead calculation. In the proposed system, synchronized limb and precordial potentials are acquired through the ADS1298 analog front-end and subsequently converted into the twelve conventional ECG leads within the host software. This configuration preserves the spatial information required for assessment of cardiac axis, repolarization abnormalities, and regional ST-segment changes.

The bipolar limb leads are defined from the electrical potentials measured at the extremity electrodes. Leads I and II are obtained directly as

$$V_{I}=V_{LA}-V_{RA},$$

$$V_{II}=V_{LL}-V_{RA},$$

where $V_{RA},\;V_{LA}$ and $V_{LL}$ denote the instantaneous electrical potentials recorded at the right-arm, left-arm, and left-leg electrodes, respectively.

According to Einthoven’s law, Lead III is reconstructed from Leads I and II as

$$V_{III}=V_{LL}-V_{LA}=V_{II}-V_{I}$$

The augmented unipolar limb leads are subsequently calculated as

$$V_{aVR}=V_{RA}-\frac{V_{LA}+V_{LL}}{2}=-\frac{V_{I}+V_{II}}{2},$$

$$V_{aVL}=V_{LA}-\frac{V_{RA}+V_{LL}}{2}=V_{I}-\frac{V_{II}}{2},$$

$$V_{aVF}=V_{LL}-\frac{V_{RA}+V_{LA}}{2}=V_{II}-\frac{V_{I}}{2}.$$

The six precordial leads represent cardiac electrical activity in the horizontal plane. Each chest electrode is referenced to the Wilson central terminal, defined as the average potential of the three limb electrodes:

$$V_{WCT}=\frac{V_{RA}+V_{LA}+V_{LL}}{3}$$

Accordingly, each precordial lead is calculated as

$$V_{VK}=V_{CK}-V_{WCT},k=1,\ldots ,6,$$

where $V_{CK}$ denotes the potential measured at the corresponding chest electrode.

All acquired channels are sampled synchronously, which is essential for preserving temporal alignment among the reconstructed leads. Lead calculations are performed sample by sample; therefore, for the *n*-th sample, each derived lead is computed from limb-electrode measurements acquired at the same sampling instant. This prevents interlead timing offsets and enables reliable comparison of QRS morphology and ST-segment deviation across anatomically contiguous leads.

Before reconstruction, the acquired channels undergo calibration and conversion from analog-to-digital converter output codes into voltage units. The general conversion can be represented as

$$V_{i}\left[n\right]=\frac{D_{i}\left[n\right]}{2^{N-1}-1}\cdot \frac{V_{ref}}{G}$$

For the 24-bit ADS1298, *N* = 24, and $D_{i}\left[n\right]$ denotes the signed two’s-complement output code. The calibrated signals are expressed in microvolts and converted to millivolts when required:

$$V_{mV}=\frac{V_{\mu V}}{1000}$$

For conventional ECG display at a calibration of 10 mm/mV, the vertical displacement is determined as

$$A_{mm}={10V}_{mV}$$

The reconstructed twelve-lead signals are stored in the following standardized order:

I, II, III, aVR, aVL, aVF, V1, V2, V3, V4, V5, V6.

This standardized representation is subsequently used for real-time visualization, interval measurement, waveform analysis, and spatial evaluation of ST-segment abnormalities. In particular, the availability of synchronized frontal- and horizontal-plane leads enables the software to assess deviations in anatomically contiguous lead groups associated with anterior, inferior, and lateral myocardial territories. The platform documentation specifies that all twelve reconstructed leads are processed by the server-side analysis pipeline and used for ST-segment and topographic assessment.

#### 2.3.5. ECG Feature Extraction

Following beat segmentation, clinically relevant electrocardiographic features are extracted from each reconstructed ECG lead. These features provide quantitative information regarding cardiac depolarization, repolarization, and ventricular conduction, and constitute the basis for subsequent rhythm analysis, ST-segment evaluation, and automatic ECG interpretation. To improve measurement robustness, feature extraction is performed on each cardiac cycle individually, followed by statistical aggregation across all detected beats.

The detected R-peaks serve as temporal reference points for localization of the remaining ECG waveform components. Using predefined search windows relative to each R-peak, the onset and offset of the P wave, QRS complex, ST segment, and T wave are identified. These fiducial points enable accurate measurement of standard ECG intervals and waveform amplitudes while preserving temporal consistency across all reconstructed leads.

The RR interval is determined from consecutive R-peaks as described in Section 2.3. Subsequently, the principal ECG intervals are measured for each cardiac cycle. The PR interval is calculated as the duration between the onset of the P wave and the onset of the QRS complex

$$PR=t_{{QRS}_{on}}-t_{P_{on}}$$

where $t_{P_{on}}$ denotes the onset of the P wave and $t_{{QRS}_{on}}$ represents the beginning of ventricular depolarization.

The duration of the QRS complex is determined as

$$QRS=t_{{QRS}_{off}}-t_{{QRS}_{on}}$$

where $t_{{QRS}_{off}}$ is the end of the QRS complex.

The QT interval is measured from the onset of ventricular depolarization to the end of ventricular repolarization

$$QT=t_{T_{off}}-t_{{QRS}_{on}}$$

where $t_{T_{off}}$ denotes the end of the T wave.

Since the QT interval varies with heart rate, corrected QT (QTc) values are additionally calculated. In the proposed system, both Bazett’s and Fridericia’s correction methods are implemented:

$${QTc}_{Bazzet}=\frac{QT}{\sqrt{RR}},$$

$${QTc}_{Fridericia}=\frac{QT}{\sqrt[{3}]{\sqrt{RR}}},.$$

Median values of the measured PR, QRS, QT, and QTc intervals across all valid cardiac cycles are subsequently used for further analysis, reducing the influence of occasional measurement errors and motion artifacts.

In addition to interval estimation, waveform amplitudes are measured relative to the local baseline defined by the PR segment. The median amplitudes of the R wave and T wave are calculated for each reconstructed lead. These measurements provide additional information regarding ventricular depolarization and repolarization and support subsequent detection of pathological ECG patterns.

The presence of a pathological Q wave is evaluated according to conventional electrocardiographic criteria. A Q wave is considered pathological if either its depth exceeds 25% of the corresponding R-wave amplitude or its duration exceeds 40 ms while maintaining a depth greater than 10% of the R-wave amplitude. These conditions are expressed as

$$Q_{depth}\geq 0.25\left|R\right|\mathrm{or}\;Q_{duration}\geq 40\;\mathrm{ms}\;\mathrm{and}\;Q_{depth}>0.10\left|R\right|.$$

T-wave polarity is assessed using the median T-wave amplitude measured within the ST–T interval. Negative median amplitudes indicate T-wave inversion. Because inverted T waves may represent normal physiological findings in selected leads, inversions observed in leads aVR, V1, and III are not considered pathological during automatic interpretation.

To improve measurement stability, feature extraction is performed for every detected cardiac cycle, after which median values are calculated across all accepted beats. This strategy minimizes the influence of isolated artifacts, ectopic beats, and occasional detection errors while providing robust estimates of clinically relevant ECG parameters for subsequent ST-segment analysis and rhythm classification.

**Figure 7:**
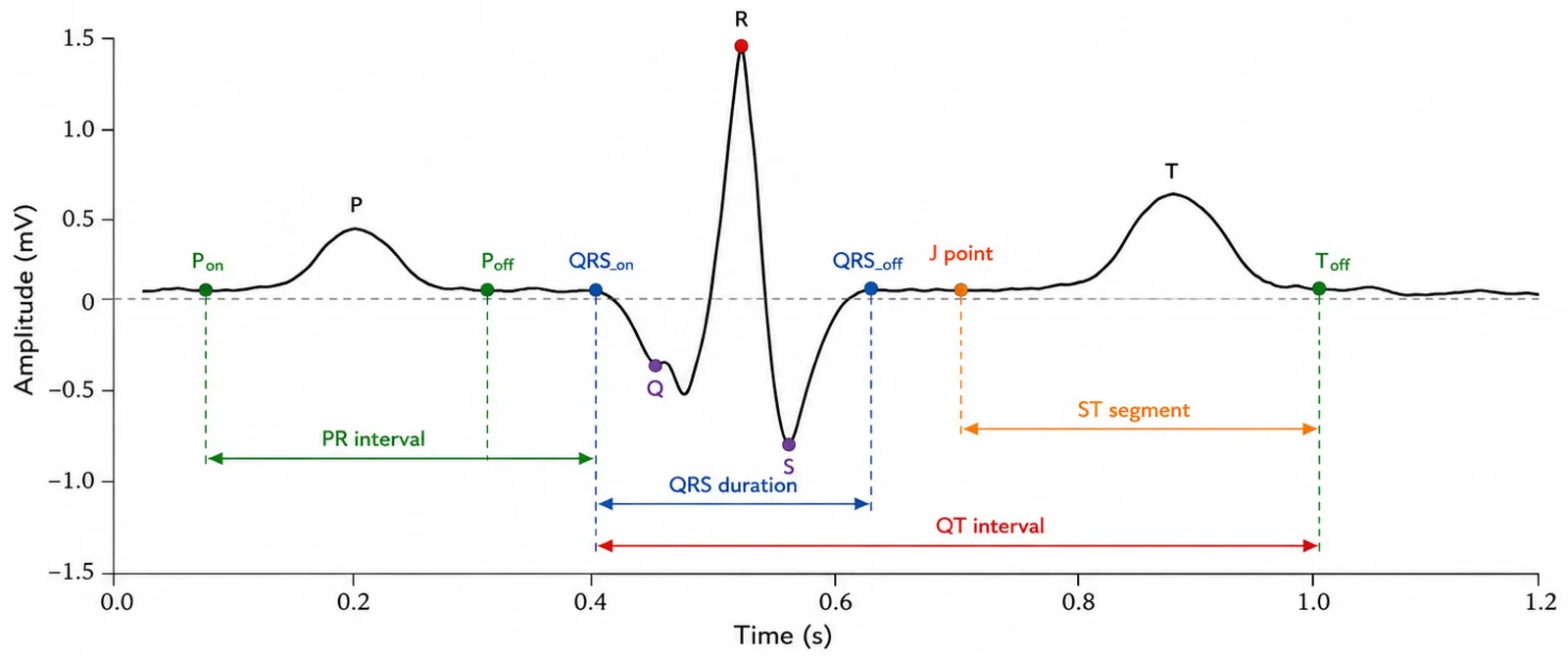
ECG feature extraction and interval measurement. Representative cardiac cycle illustrating the fiducial points automatically identified by the proposed algorithm, including the onset and offset of the P wave, QRS complex, J point, T-wave offset, and the corresponding PR interval, QRS duration, QT interval, and ST segment. The Q point is defined as the local minimum of the negative deflection preceding the R wave and is distinguished from QRS onset, which represents the beginning of the QRS complex.

To facilitate visualization of the extracted ECG features, the principal fiducial points and measured intervals within a representative cardiac cycle are illustrated in Figure 7. The figure demonstrates the locations of the P wave, QRS complex, ST segment, T wave, and the corresponding temporal intervals used during automatic feature extraction.

The extracted fiducial points serve as the basis for quantitative measurement of clinically relevant ECG parameters. Accurate localization of the P wave, QRS complex, J-point, and T wave enables reliable estimation of cardiac intervals and waveform amplitudes, which are subsequently utilized for ST-segment evaluation and rhythm analysis. Because all measurements are performed using synchronized twelve-lead recordings, temporal consistency is preserved across all ECG leads, thereby improving the reliability of multilead morphological analysis.

To reduce the influence of occasional detection errors and motion artifacts, all interval and amplitude measurements are calculated for individual cardiac cycles and subsequently summarized using their median values. This strategy improves the robustness of the extracted ECG parameters and provides stable input for the automatic interpretation algorithms described in the following section.

#### 2.3.6. ST-Segment Analysis

The ST segment represents the period between ventricular depolarization and repolarization and constitutes one of the most clinically significant regions of the electrocardiogram for the diagnosis of acute myocardial ischemia and infarction. Accurate measurement of ST-segment deviation is therefore an essential component of automated ECG interpretation systems. Because ST-segment morphology is highly sensitive to baseline fluctuations, reliable estimation requires accurate localization of the J-point together with robust baseline correction and precise delineation of the cardiac cycle.

In the proposed system, ST-segment analysis is performed after completion of ECG preprocessing, R-peak detection, beat segmentation, and twelve-lead reconstruction. For each detected cardiac cycle, the J-point is identified as the end of the QRS complex, marking the transition between ventricular depolarization and the ST segment. The isoelectric reference level is estimated from the PR segment, which is considered electrically stable under normal physiological conditions.

The ST-segment deviation is measured at a fixed interval following the J-point. In accordance with current clinical recommendations, the ST amplitude is evaluated 80 ms after the J-point (ST80), providing a stable reference point for assessment of myocardial injury.

The ST-segment deviation is calculated as

$${ST}_{80}=V\left(J+80\;\mathrm{ms}\right)-V_{baseline}$$

where $V\left(J+80\;\mathrm{ms}\right)$ denotes the ECG amplitude measured 80 ms after the J-point and $V_{baseline}$ represents the mean voltage of the PR segment.

For each reconstructed ECG lead, ST-segment deviation is determined independently. To improve measurement robustness, the median ST deviation is calculated across all accepted cardiac cycles:

$${ST}_{midian}=median\left({ST}_{1},{ST}_{2},\;\ldots ,\;{ST}_{n}\right)$$

where ${ST}_{i}$ represents the ST-segment deviation measured for the i-th cardiac cycle.

Because myocardial ischemia typically affects anatomically contiguous regions of the myocardium, the proposed software evaluates ST-segment deviations across groups of contiguous ECG leads rather than considering each lead independently. The analyzed lead groups include:

Inferior leads: II, III, aVF;

Lateral leads: I, aVL, V5, V6;

Anterior leads: V1–V4.

Simultaneous ST-segment elevation or depression observed within contiguous lead groups increases the diagnostic confidence of automated interpretation and reduces the influence of isolated measurement artifacts.

To prevent false-positive detections caused by noise or isolated abnormal beats, ST-segment analysis is performed only for cardiac cycles that satisfy the signal quality criteria described in Section 2.3.1. Furthermore, measurements are excluded when excessive baseline instability or abnormal beat morphology is detected.

The calculated ST-segment deviations from all twelve reconstructed ECG leads constitute one of the principal diagnostic inputs to the automatic ECG interpretation module. These measurements are subsequently integrated with rhythm analysis and interval estimation to generate a preliminary clinical interpretation while preserving physician oversight during the final diagnostic decision.

**Figure 8:**
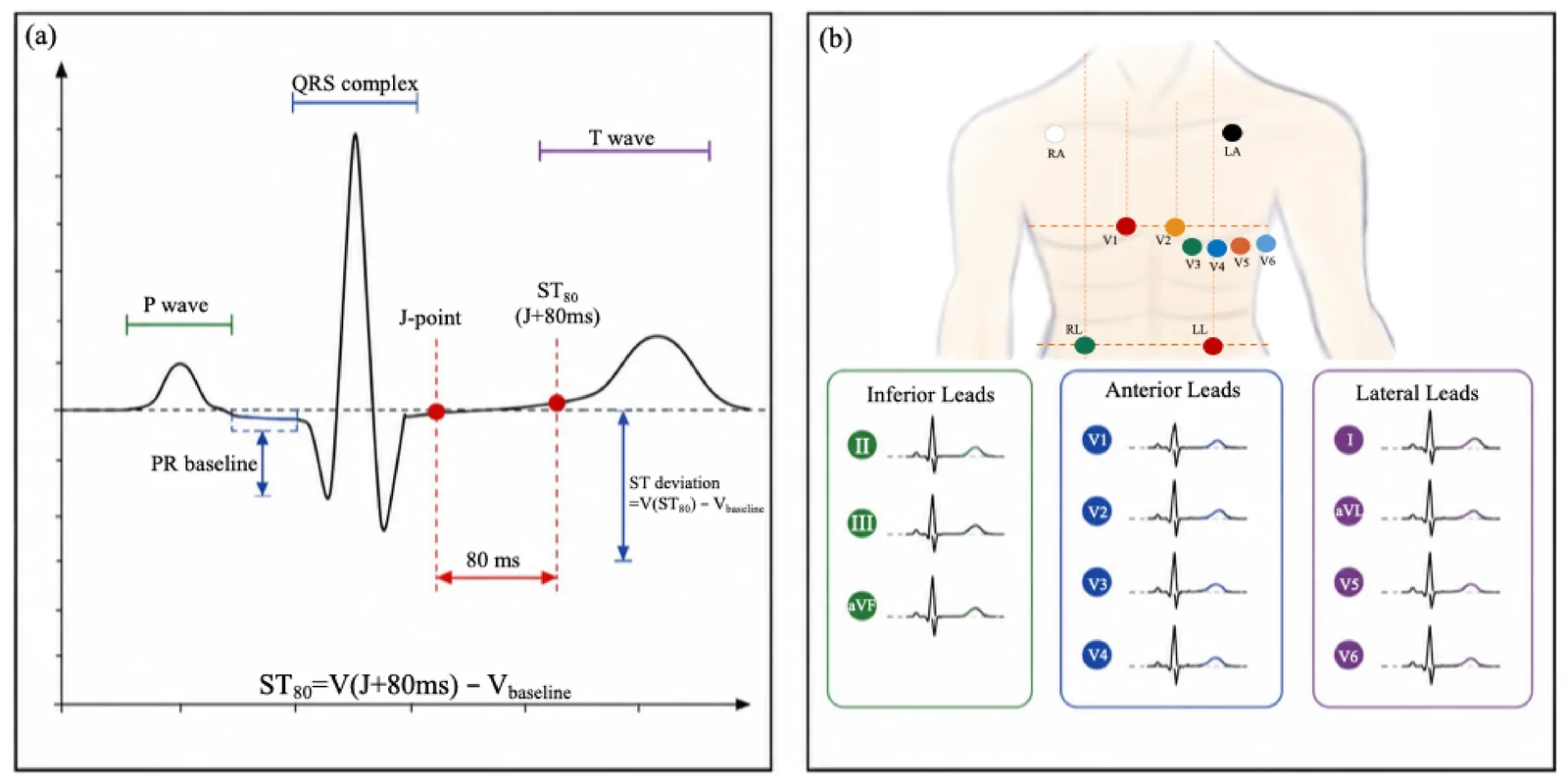
ST-segment deviation measurement and anatomically contiguous lead groups used for regional assessment of myocardial ischemia. (**a**) ST_80_ measurement relative to the PR-segment isoelectric baseline. (**b**) Anatomically contiguous ECG lead groups used for regional assessment of ST-segment abnormalities.

The measurement of ST-segment deviation implemented in the proposed software platform is illustrated in Figure 8. The algorithm determines the isoelectric reference level from the PR segment and identifies the J-point as the transition between the QRS complex and the ST segment. The ST amplitude is subsequently measured 80 ms after the J-point (ST_80_) for each reconstructed ECG lead. In addition to single-lead measurements, the software evaluates ST-segment deviations across anatomically contiguous lead groups to improve the reliability of myocardial ischemia detection.

As shown in Figure 8a, the ST-segment deviation is determined as the voltage difference between the ST_80_ measurement point and the isoelectric baseline estimated from the PR segment. This approach minimizes the influence of baseline drift and provides consistent estimation of ST-segment displacement across all reconstructed ECG leads.

The regional distribution of ST-segment deviations is subsequently analyzed using anatomically contiguous lead groups, as illustrated in Figure 8b. Simultaneous ST elevation or depression observed in multiple adjacent leads increases the diagnostic confidence for myocardial ischemia while reducing false-positive detections caused by isolated artifacts or measurement errors. The resulting ST-segment measurements are integrated with the extracted ECG features and rhythm analysis to generate the preliminary diagnostic interpretation described in the subsequent section.

#### 2.3.7. Myocardial Infarction Detection

The final stage of the proposed software framework performs automated detection of electrocardiographic patterns suggestive of acute myocardial infarction (MI). The diagnostic algorithm integrates the results of signal preprocessing, twelve-lead reconstruction, feature extraction, and ST-segment analysis to generate a preliminary clinical interpretation intended to assist physicians during ECG assessment. The algorithm is designed as a clinical decision-support tool and does not replace expert medical diagnosis.

The primary diagnostic criterion is the analysis of ST-segment deviation measured at the ST_80_ point for each reconstructed ECG lead. After baseline correction and ST_80_ measurement, every lead is independently classified into one of four categories according to the magnitude of ST deviation:

Normal;

Borderline;

ST elevation;

ST depression.

The classification thresholds implemented in the software are summarized in Table 5. An ST deviation greater than or equal to 0.10 mV (1 mm) is considered clinically significant, whereas deviations between 0.05 mV and 0.10 mV are classified as borderline findings. These thresholds are consistent with the values implemented in the signal analysis module.

Following per-lead classification, the algorithm evaluates anatomically contiguous lead groups corresponding to the principal myocardial territories. Three regional groups are analyzed:

Anterior wall: V1–V4;

Inferior wall: II, III, aVF;

Lateral wall: I, aVL, V5, and V6.

For each myocardial territory, the software searches for consistent ST-segment elevation or depression across adjacent ECG leads. Regional abnormalities are considered clinically significant only when similar changes are simultaneously detected in at least two contiguous leads, thereby reducing false-positive detections caused by isolated artifacts or local measurement errors.

In addition to ST-segment analysis, the diagnostic algorithm incorporates complementary electrocardiographic features extracted during the previous processing stages, including pathological Q waves, T-wave inversion, heart rate, PR interval, QRS duration, QT/QTc intervals, and signal quality assessment. These parameters provide supportive diagnostic information and improve the reliability of automatic interpretation, particularly in recordings containing borderline ST-segment abnormalities.

The preliminary diagnostic report is generated by combining regional ST-segment analysis with the extracted ECG features. When ST elevation is detected in two or more contiguous leads within the same myocardial territory, the software identifies a ST-elevation myocardial infarction (STEMI) pattern and reports the suspected localization of myocardial injury (anterior, inferior, or lateral wall). The generated report also summarizes measured ECG parameters, wave morphology, and signal quality, providing physicians with a structured overview of the automated analysis while preserving clinical responsibility for the final diagnosis. The report clearly indicates that the automatically generated conclusion is preliminary and requires confirmation by a qualified cardiologist.

#### 2.3.8. Statistical Agreement Analysis

Quantitative agreement between measurements obtained using the CardioService software and the reference BTL Flexi 12 ECG was evaluated in 30 paired recordings. The primary interval-based analysis included PR interval, QRS duration, QT interval, and heart-rate-corrected QT interval (QTc), with measurements compared between the two systems on a patient-by-patient basis. In addition, a lead-specific analysis was performed for R-wave and T-wave amplitudes in each of the twelve ECG leads (I, II, III, aVR, aVL, aVF, and V1–V6). For the reference system, the corresponding lead-specific amplitude measurements were retrieved from the patient examination records stored in the BTL Flexi 12 ECG software and paired with the CardioService measurements for the same participants and leads.

Agreement was characterized using the mean absolute error (MAE), root mean square error (RMSE), and mean paired difference (bias), calculated as the CardioService measurement minus the corresponding BTL reference measurement. Linear association was assessed using Pearson’s correlation coefficient (r). Because correlation alone does not quantify agreement between measurement methods, intraclass correlation coefficients (ICC) were additionally calculated for the lead-specific R-wave and T-wave amplitude measurements using a two-way random-effects, absolute-agreement, single-measure model [ICC(2,1)]. Bland–Altman analysis was performed to estimate the 95% limits of agreement (LoA), calculated as the mean paired difference ± 1.96 standard deviations of the paired differences.

#### 2.3.9. Automatic ECG Interpretation

The final stage of the proposed software framework performs automatic interpretation of the reconstructed twelve-lead electrocardiogram by integrating the results obtained during all preceding processing stages. Rather than relying on a single electrocardiographic parameter, the interpretation algorithm combines signal quality assessment, interval measurements, waveform morphology, ST-segment evaluation, rhythm analysis, and regional myocardial localization to generate a comprehensive preliminary diagnostic report. The generated interpretation is intended exclusively as a clinical decision-support tool and does not replace physician judgment.

Initially, the software evaluates the quality of each ECG recording to ensure reliable interpretation. Recordings with excessive noise, poor electrode contact, or unstable baseline characteristics are identified during the signal quality assessment stage. Only ECG recordings satisfying predefined quality criteria are considered suitable for subsequent diagnostic analysis, thereby reducing the probability of false-positive findings arising from poor signal quality.

Following quality verification, the extracted electrocardiographic parameters are integrated into a rule-based interpretation engine. The principal diagnostic features include heart rate, PR interval, QRS duration, QT/QTc intervals, ST_80_ deviation, pathological Q waves, T-wave inversion, and rhythm regularity. These parameters are analyzed simultaneously to evaluate both electrical conduction abnormalities and myocardial ischemic changes.

Automatic interpretation of myocardial ischemia is primarily based on regional ST-segment analysis. After classification of individual leads as normal, borderline, ST elevation, or ST depression, the software evaluates contiguous lead groups corresponding to the anterior, inferior, and lateral myocardial territories. Detection of significant ST elevation in two or more contiguous leads within the same anatomical region results in automatic identification of a ST-elevation myocardial infarction (STEMI) pattern, together with estimation of the probable localization of myocardial injury.

Besides ischemia detection, the interpretation algorithm evaluates additional electrocardiographic abnormalities. Pathological Q waves and T-wave inversion are incorporated as supportive indicators of myocardial injury, whereas rhythm analysis identifies sinus rhythm, bradycardia, tachycardia, rhythm irregularity, suspected atrial fibrillation, significant pauses, and premature ventricular contractions according to the implemented decision rules. These complementary findings increase the robustness of automatic interpretation while providing clinically meaningful information for physician review.

The final interpretation is presented as a structured clinical report containing quantitative ECG measurements, lead-specific ST-segment evaluation, myocardial territory analysis, detected rhythm abnormalities, waveform morphology, and an automatically generated preliminary conclusion. The software additionally reports the estimated signal quality to assist physicians in judging the reliability of the analysis. An example of the automatically generated interpretation includes identification of sinus rhythm, localization of ST-segment elevation within anterior leads (V1–V3), recognition of a suspected STEMI pattern, detection of pathological Q waves and T-wave inversion, and notification that the result requires confirmation by a qualified cardiologist before clinical use.

The proposed interpretation framework therefore provides comprehensive automated ECG assessment while maintaining full physician supervision over the final diagnostic decision. By integrating multiple electrocardiographic features rather than relying on isolated measurements, the software improves the consistency of preliminary ECG interpretation and facilitates rapid clinical assessment in telemedicine and emergency care environments. The diagnostic features integrated into the automatic ECG interpretation module are summarized in Table 6.

## 3. Results

The proposed third-generation wearable ECG platform was evaluated with respect to its technical implementation, ability to acquire and reconstruct the standard twelve-lead electrocardiogram, quality and clinical interpretability of the recorded signals, comparability with a stationary reference electrocardiograph, and feasibility of automated multilead ST-segment analysis. The results are presented according to the principal objectives defined for the study: architectural optimization of the wearable platform, technical and clinical evaluation of twelve-lead ECG acquisition, and demonstration of spatial ECG analysis and automated preliminary interpretation.

A prospective clinical evaluation with elements of comparative analysis was conducted at the Research Institute of Cardiology and Internal Medicine, Almaty, Kazakhstan. The study included 30 participants aged 18–56 years (mean age, 26.2 ± 9.9 years), comprising 25 men (83.3%) and 5 women (16.7%). None of the participants had a known history of cardiovascular disease at the time of enrollment. The mean height and body weight were 1.77 ± 0.09 m and 73.8 ± 13.0 kg, respectively, with a mean body mass index (BMI) of 23.8 ± 4.6 kg/m^2^. Information on smoking status, alcohol consumption, and physical activity was also collected. The demographic, anthropometric, and lifestyle characteristics of the study participants are summarized in Table 7.

ECG recordings obtained using the proposed wearable system were evaluated against recordings acquired using a BTL Flexi 12 ECG clinical electrocardiograph (BTL Industries Ltd., Hertfordshire, United Kingdom), which served as the reference system. The recordings with the two systems were acquired sequentially rather than simultaneously, with the electrodes repositioned between examinations according to the standard ten-electrode configuration. Consequently, differences in electrode placement, particularly for the precordial leads, could not be completely excluded. The ECG recordings were assessed at a display speed of 25 mm/s and a sensitivity of 10 mm/mV.

### 3.1. Technical Implementation and System Performance

The third-generation wearable ECG platform was successfully implemented using an integrated ESP32-S3 system-on-chip architecture. The controller provided centralized management of synchronized ECG acquisition, communication with the ADS1298 analog front-end, local data storage, OLED visualization, wireless transmission, and device-level control. Integration of these functions within a single controller eliminated the separate STM32, ESP32 communication, and HC-05 Bluetooth modules used in the preceding platform generation.

The proposed PCB measured 60 × 80 mm, compared with 60 × 100 mm for the preceding multilead platform. The reduction in board height from 100 to 80 mm resulted in a 20% decrease in PCB area while preserving the ten-electrode interface and twelve-lead ECG capability. This dimensional reduction facilitated a more compact device enclosure and reduced the number of intermodule electrical connections. The hardware redesign primarily involved consolidation of functions previously distributed across multiple controller and communication modules rather than modification of the underlying ECG acquisition principle. Integration of these functions within the ESP32-S3 architecture reduced the number of intermodule connections and communication interfaces and contributed to the reduction in PCB dimensions, while the ten-electrode configuration and synchronized multichannel acquisition were retained.

The system maintained synchronized multichannel acquisition at a sampling frequency of 500 Hz. During clinical recordings, the device supported continuous acquisition, local microSD storage, and transfer of ECG data to the host application. Complete twelve-lead recordings were obtained in all evaluated cases, indicating reliable operation of the acquisition, reconstruction, and data-management subsystems under standard clinical recording conditions.

The measured current consumption of the integrated platform was approximately 30 mA, corresponding to a power consumption of approximately 0.1 W under the tested operating configuration. These results demonstrate that the architectural integration achieved reduced board dimensions and system complexity while retaining the functional resources required for real-time multilead ECG processing.

The measured technical characteristics summarized in Table 8 confirm that the proposed architecture retained the multilead functionality of the preceding platform while achieving a more compact and integrated hardware implementation.

### 3.2. Clinical Evaluation and Twelve-Lead Recording Quality

During the prospective clinical evaluation, the proposed wearable system successfully acquired complete twelve-lead ECG recordings in all 30 participants. The standard leads I, II, III, aVR, aVL, aVF, and V1–V6 were correctly formed using the conventional ten-electrode configuration. No recording was excluded because of failure to generate the complete twelve-lead set.

Signal quality was rated as good in at least 90% of the evaluated recordings. No substantial artifacts capable of preventing clinical interpretation were reported. All recordings were considered sufficiently readable for assessment of heart rate, cardiac rhythm, conduction intervals, waveform morphology, and repolarization characteristics.

Expert visual assessment included baseline stability, noise level, power-line interference, motion artifacts, clarity of waveform boundaries, completeness of the twelve-lead recording, and continuity of signal acquisition. The recordings demonstrated stable baselines and sufficiently low artifact levels under standard clinical conditions. Where minor noise was present, the P wave, QRS complex, and T wave remained distinguishable.

The clinical evaluation therefore demonstrated that the proposed system was technically capable of acquiring interpretable twelve-lead ECG signals across all enrolled participants. Nevertheless, because the study was designed as a pilot clinical evaluation, the findings should be interpreted as preliminary and require confirmation in a larger and more heterogeneous cohort. A summary of the clinical evaluation and signal-quality assessment is provided in Table 9.

### 3.3. Morphological and Quantitative Comparison with the Reference ECG

The principal morphological components of the ECG were clearly visualized in the wearable recordings. Expert assessment confirmed identifiable P waves, QRS complexes, and T waves without clinically significant deformation. The reconstructed signals retained sufficient temporal and amplitude information for assessment of cardiac rhythm, conduction, and repolarization.

Qualitative review confirmed that the principal ECG components, including the P wave, QRS complex, and T wave, were identifiable in recordings obtained with the proposed system. However, direct waveform-level morphological equivalence with the BTL reference electrocardiograph could not be established because the recordings were acquired sequentially with electrode repositioning between examinations. Differences in precordial QRS and T-wave morphology were observed, particularly in leads V2–V4. Accordingly, the visual comparison was used to assess the interpretability and completeness of the twelve-lead recordings rather than to demonstrate morphological interchangeability between the two systems.

In addition to qualitative morphological assessment, quantitative agreement between the CardioService software and the reference BTL Flexi 12 ECG was evaluated for automatically determined PR, QRS, QT, and QTc intervals across all 30 paired recordings.

Moderate positive correlations were observed for the evaluated interval parameters, with Pearson correlation coefficients ranging from r = 0.756 to r = 0.820 (all *p* < 0.001). Because Pearson correlation reflects association rather than agreement, these coefficients were interpreted together with the MAE, RMSE, bias, and Bland–Altman limits of agreement and were not used to infer interchangeability between the two systems. The mean absolute errors were 7.00 ms for PR, 2.53 ms for QRS duration, 10.40 ms for QT, and 9.57 ms for QTc. Mean paired differences were −0.73 ms, +0.93 ms, −0.73 ms, and −3.30 ms for PR, QRS, QT, and QTc, respectively. Bland–Altman analysis yielded 95% limits of agreement of −17.10 to 15.63 ms for PR, −5.86 to 7.72 ms for QRS, −25.83 to 24.36 ms for QT, and −23.23 to 16.63 ms for QTc. The quantitative comparison showed measurable agreement between the CardioService and BTL measurements, with the corresponding errors, biases, and limits of agreement reported in Table 10.

Bias was calculated as CardioService—BTL Flexi 12 ECG. All Pearson correlations were statistically significant (*p* < 0.001). LoA, limits of agreement; MAE, mean absolute error; RMSE, root mean square error.

To further examine the lead-dependent differences observed during morphological assessment, a lead-specific quantitative comparison was performed for R-wave and T-wave amplitudes across all 30 paired recordings. R-wave amplitude showed consistently high association between the two systems in most leads, with Pearson correlation coefficients ranging from 0.857 to 0.992. The corresponding ICC values ranged from 0.772 to 0.991. The lowest R-wave agreement was observed in V4 (r = 0.857; ICC = 0.772), followed by V3 (r = 0.898; ICC = 0.856).

Greater lead-dependent variation was observed for T-wave amplitude. Limb leads and leads V1, V5, and V6 generally showed higher agreement, whereas the anterior precordial leads V2–V4 showed lower agreement. In V2, V3, and V4, Pearson correlation coefficients for T-wave amplitude were 0.805, 0.716, and 0.727, respectively, while the corresponding ICC values were 0.513, 0.446, and 0.475. These findings in the Table 11 quantitatively support the lead-dependent morphological differences observed in the precordial leads and indicate that the two systems should not be considered morphologically interchangeable on the basis of the present dataset.

ICC, intraclass correlation coefficient. ICC values were calculated using a two-way random-effects, absolute-agreement, single-measure model [ICC(2,1)]. Pearson correlation coefficients describe linear association, whereas ICC values were used to characterize absolute agreement between the two measurement systems. Lead-specific Bland–Altman analysis was also performed for R-wave and T-wave amplitudes; the corresponding biases and 95% limits of agreement are provided in Supplementary Table S1.

**Figure 9:**
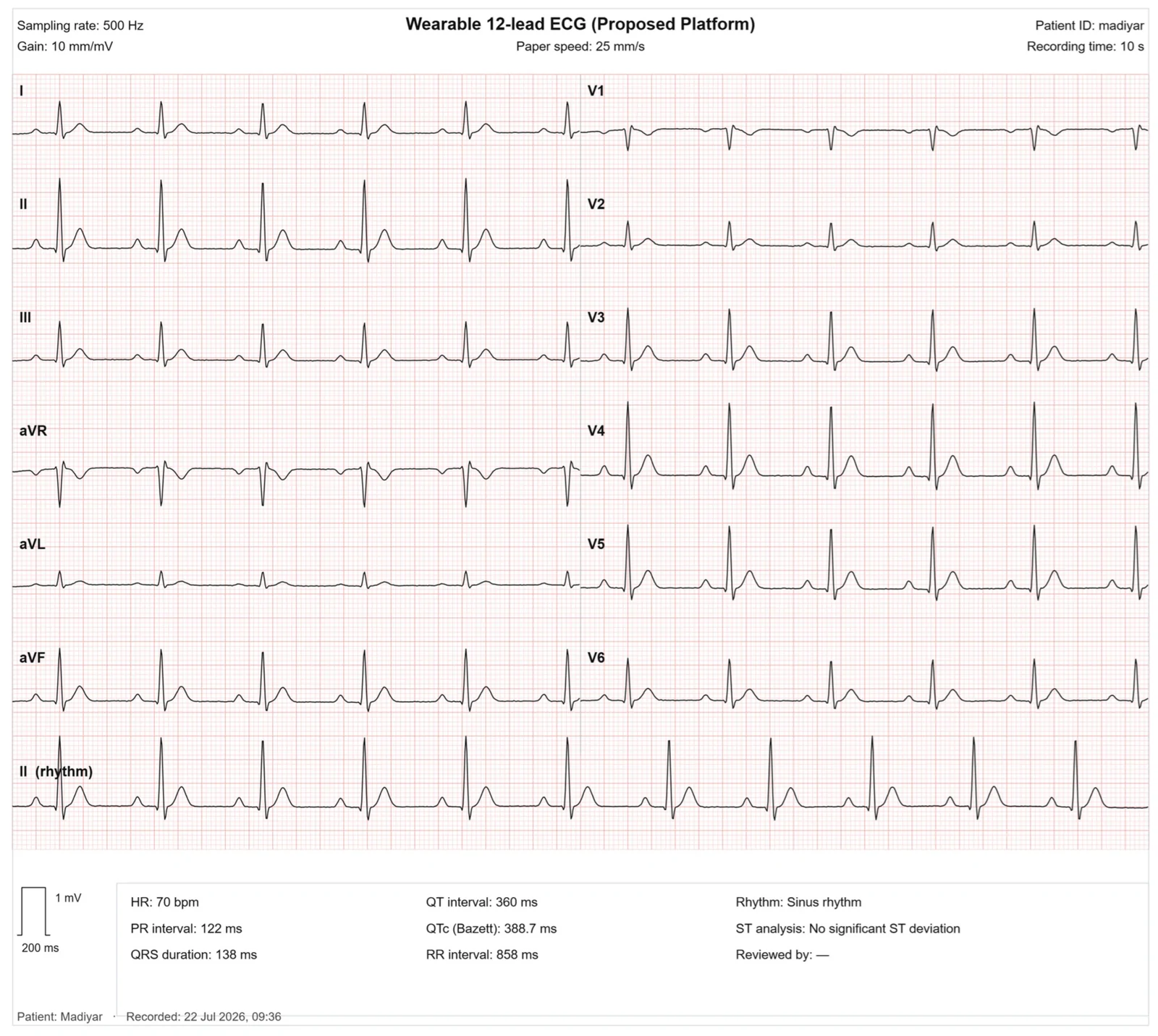
Representative twelve-lead ECG recorded using the proposed wearable platform.

A representative twelve-lead ECG acquired with the proposed wearable platform is shown in Figure 9.

**Figure 10:**
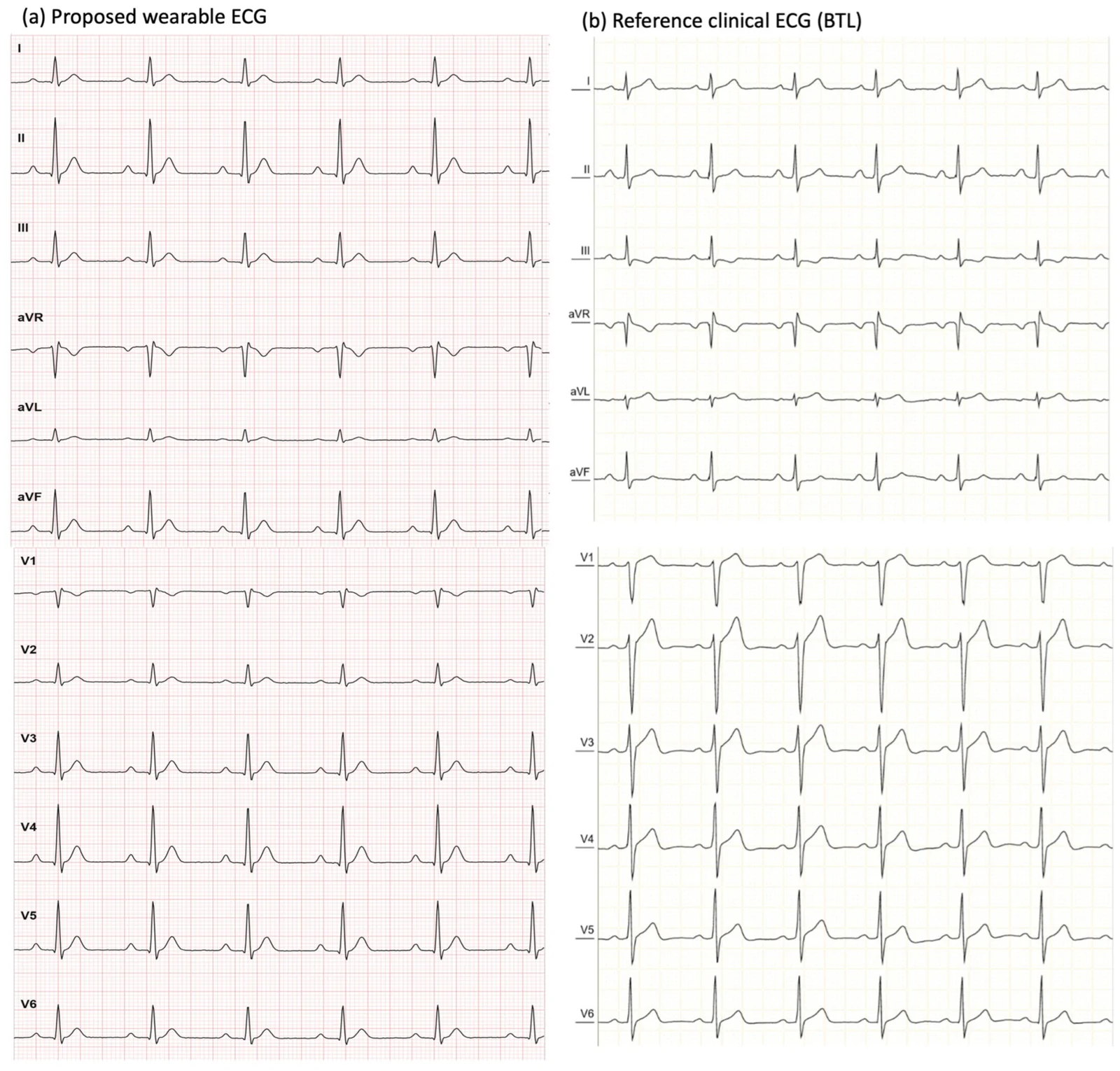
Representative twelve-lead ECG recordings obtained using (**a**) the proposed wearable ECG platform and (**b**) the reference clinical electrocardiograph (BTL). The recordings were acquired sequentially with electrode repositioning between examinations. Differences in QRS and T-wave morphology are visible in the precordial leads, particularly V2–V4; therefore, the recordings are not presented as evidence of waveform-level morphological equivalence between the two systems.

Figure 10 presents representative twelve-lead ECG recordings obtained using the proposed wearable platform and the reference clinical electrocardiograph. The recordings were acquired sequentially rather than simultaneously, with electrode repositioning between the two examinations. Visual comparison shows differences in QRS and T-wave morphology in the precordial leads, particularly V2–V4. Because the recordings were obtained at different times and the electrodes were repositioned, the figure is presented as an illustrative comparison of twelve-lead ECG acquisition and should not be interpreted as evidence of waveform-level morphological equivalence between the two systems.

Although the present study primarily focused on technical validation rather than diagnostic accuracy, comparison with the reference clinical electrocardiograph confirmed the ability of the proposed system to acquire and display complete twelve-lead ECG recordings. Direct morphological equivalence between the two systems was not established in the present study. The principal technical and functional characteristics of the proposed platform and the reference clinical electrocardiograph are compared in Table 12.

To contextualize the quantitative performance of the developed CardioService software, the obtained interval-measurement results were compared with previously reported contemporary ECG analysis and validation approaches. Because these studies differ in ECG configuration, study population, reference method, and statistical methodology, the comparison is intended to provide a performance context rather than a direct ranking of the systems (Table 13).

### 3.4. Multilead ST-Segment Analysis

The automated signal-processing module successfully calculated lead-specific ST-segment deviation for each of the twelve reconstructed ECG leads. Each lead was classified as normal, borderline, elevated, or depressed according to the predefined amplitude thresholds. The software subsequently evaluated anatomically contiguous lead groups corresponding to the anterior, inferior, and lateral myocardial territories.

A representative recording demonstrated ST-segment elevation in leads V1, V2, and V3, with measured deviations of 0.194, 0.262, and 0.163 mV, respectively. Lead V4 showed a borderline deviation of 0.080 mV, whereas the remaining leads were classified as normal. The software therefore localized the predominant ST-segment abnormality to the anterior lead group.

These findings demonstrate the technical feasibility of automated spatial ST-segment assessment using the reconstructed twelve-lead ECG. In addition to the interval-based validation, lead-specific quantitative agreement was assessed for R-wave and T-wave amplitudes across all 30 paired recordings. Direct paired validation of ST_80_, however, was not performed because the lead-specific ST-level values stored by the BTL system could not be confirmed to use the same J + 80 ms measurement point implemented in CardioService. Comparing these values directly could therefore involve non-equivalent measurement definitions. The ST_80_ and regional ischemia-analysis functions were evaluated at the present stage as a proof of technical feasibility rather than as a diagnostic-accuracy study. Because the enrolled cohort did not include patients with confirmed cardiovascular disease, sensitivity, specificity, and predictive values for ischemia or myocardial infarction detection could not be reliably estimated. Accordingly, validation of the automated ST-segment classification and myocardial-territory localization against expert-annotated pathological ECG recordings is planned as a subsequent stage of the study. The system was able to evaluate individual leads, identify consistent abnormalities across contiguous leads, and associate the observed distribution with a probable myocardial territory. This case should be interpreted as a functional demonstration of the analysis pipeline rather than evidence of diagnostic sensitivity or specificity for myocardial infarction. The lead-specific ST-segment measurements for the representative case are summarized in Table 14.

### 3.5. Automatic ECG Interpretation and Report Generation

The CardioService application successfully integrated the results of signal-quality assessment, R-peak detection, interval measurement, waveform analysis, rhythm evaluation, and lead-wise ST-segment assessment into a structured preliminary report.

A separately simulated ECG recording was used to demonstrate the operation of the regional ST-segment analysis algorithm. In this simulated example, the software identified ST-segment elevation in leads V1, V2, and V3, with measured deviations of 0.194, 0.262, and 0.163 mV, respectively. The overall signal quality was classified as good.

Based on the regional ST-segment distribution, the software generated a preliminary conclusion indicating sinus rhythm, ST elevation in V1–V3, possible anterior-wall involvement, and an ST-elevation pattern suspicious for STEMI. Pathological Q-wave flags and T-wave inversion were also included in the automated report. The interface explicitly indicated that the generated conclusion was preliminary and required confirmation by a cardiologist.

**Figure 11:**
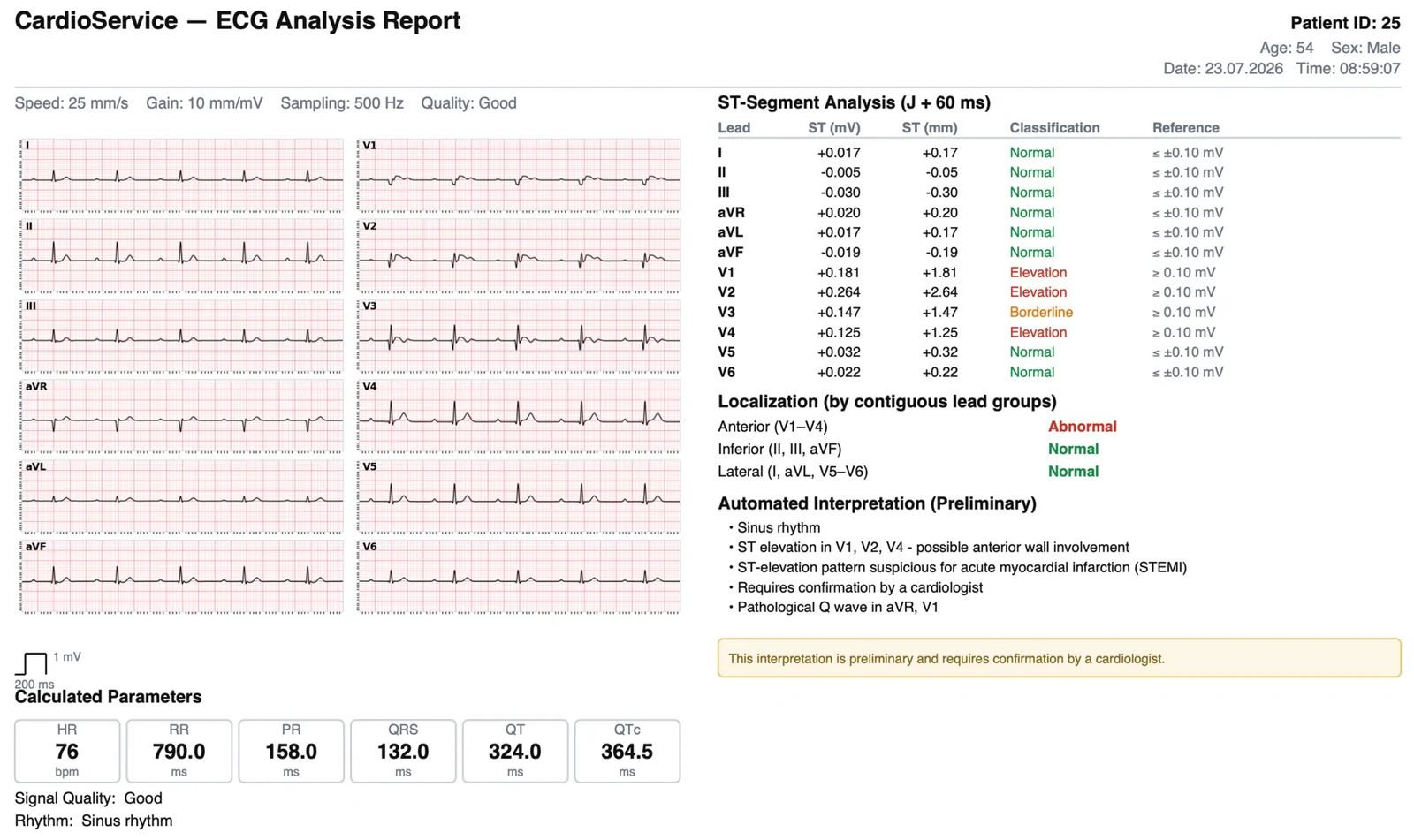
Representative output of the regional ST-segment analysis module for a simulated ECG recording with an anterior ST-elevation pattern. The example was generated to demonstrate the operation of the rule-based contiguous-lead analysis algorithm and was not derived from the clinical recordings used for comparison with the BTL reference electrocardiograph.

The successful generation of lead-specific measurements, myocardial-territory localization, and a structured preliminary conclusion demonstrates the functional integration of the acquisition and interpretation workflow. However, the diagnostic statements generated by the software should be considered decision-support outputs and not independent clinical diagnoses. A representative output of the regional ST-segment analysis module is shown in Figure 11.

## 4. Discussion

The present study demonstrated the feasibility of integrating wearable multichannel ECG acquisition, automated signal processing, and preliminary clinical interpretation within a compact third-generation telemedicine platform. The proposed system combines a miniaturized hardware architecture based on the ESP32-S3 microcontroller with synchronized twelve-lead ECG reconstruction, embedded wireless communication, automated feature extraction, multilead ST-segment analysis, and generation of a structured preliminary ECG report. Unlike conventional portable ECG recorders that primarily provide signal acquisition, the developed platform performs multiple stages of ECG analysis within a unified software ecosystem, thereby extending its functionality beyond simple physiological monitoring.

A major contribution of the proposed platform is the transition from a distributed hardware architecture to an integrated embedded system. Previous generations of the developed device relied on multiple independent microcontrollers responsible for signal acquisition, wireless communication, and peripheral control. By integrating these functions into a single ESP32-S3 system-on-chip, the overall hardware complexity was substantially reduced while maintaining synchronized multichannel acquisition, local storage, real-time wireless transmission, and visualization. The reduction in the PCB dimensions by approximately 20% further improved portability without compromising ECG acquisition quality or computational functionality. Such architectural simplification is particularly important for wearable medical devices intended for long-term ambulatory monitoring, where compact size, low power consumption, and system reliability directly influence user acceptance.

The obtained ECG recordings demonstrated stable waveform morphology and sufficient quality for clinical interpretation. During the clinical evaluation, complete twelve-lead ECG recordings were successfully obtained in all participants, and expert assessment confirmed adequate visualization of the principal ECG components, including P waves, QRS complexes, and T waves. More than 90% of the recordings were rated as good quality, indicating that the proposed acquisition hardware, electrode configuration, and preprocessing algorithms provided signals of sufficient quality for visual ECG assessment under the conditions of this pilot study. This signal-quality assessment should be distinguished from device-to-device morphological agreement, which was evaluated separately and showed lead-dependent differences, particularly in V2–V4.

Another important contribution of this work is the implementation of automated multilead ST-segment analysis using reconstructed twelve-lead ECG recordings. Most wearable ECG devices reported in the literature are limited to one- or few-lead recordings, restricting their ability to identify regional ischemic changes and localize myocardial injury. In contrast, the proposed software evaluates ST_80_ deviation independently in each reconstructed lead, classifies lead-specific abnormalities, and subsequently analyzes anatomically contiguous lead groups corresponding to the anterior, inferior, and lateral myocardial territories. This regional approach more closely reflects routine clinical interpretation of twelve-lead ECGs and provides substantially more clinically relevant information than isolated lead analysis. The automatic localization of abnormal ST-segment patterns further enhances the usefulness of the system as a decision-support tool for rapid cardiovascular assessment.

The developed software architecture also demonstrates the advantages of combining embedded acquisition with advanced desktop-based signal analysis. Separation of computationally demanding processing tasks from the wearable hardware allows implementation of robust preprocessing, feature extraction, rhythm analysis, interval measurement, and automatic report generation without increasing the computational burden of the embedded device. Such a distributed processing strategy improves system scalability and facilitates future implementation of more sophisticated machine-learning algorithms while preserving real-time ECG acquisition.

Compared with previously reported wearable ECG systems, the proposed platform provides a broader integration of hardware and software functionalities. In addition to continuous twelve-lead ECG acquisition, the system incorporates automatic ECG reconstruction, comprehensive interval measurements, multilead ST-segment evaluation, myocardial territory localization, and structured preliminary reporting within a unified telemedicine environment. This level of functional integration may improve the efficiency of remote cardiovascular assessment and facilitate rapid review of ECG recordings by healthcare professionals.

Compared with representative wearable ECG technologies and contemporary commercial portable twelve-lead electrocardiographs, the proposed platform combines multilead acquisition with a research-oriented spatial ECG analysis workflow. The principal functional differences are summarized in Table 15.

It should be emphasized that the comparison with the commercial portable twelve-lead ECG system in Table 13 is based on publicly available technical specifications and is intended to contextualize the functional scope of the proposed platform rather than to establish performance superiority. No direct head-to-head experimental comparison with the Biocare iE10 was performed in the present study. Accordingly, conclusions regarding comparative diagnostic accuracy, signal quality, or clinical performance cannot be drawn from this comparison and require dedicated validation in future studies.

The quantitative validation further demonstrated that CardioService achieved measurement errors within the range reported for contemporary ECG-analysis approaches. Particularly, the QRS measurement showed a low MAE of 2.53 ms, while the MAEs for PR and QT were 7.00 and 10.40 ms, respectively. These findings should be interpreted cautiously because of differences in populations, ECG configurations, and validation protocols among the compared studies. Nevertheless, the results support the feasibility of the developed software for automated ECG interval assessment.

As summarized in Table 11, most currently available wearable ECG technologies are primarily intended for rhythm monitoring using one or a limited number of ECG leads. Although Holter monitoring provides multilead recordings, its clinical workflow is generally based on retrospective analysis and does not inherently support integrated real-time wireless transmission or automated spatial ECG interpretation. In contrast, the proposed platform combines synchronized twelve-lead ECG acquisition, automatic ECG reconstruction, multilead ST_80_ assessment, contiguous-lead evaluation, myocardial territory localization, and generation of a structured preliminary report within a single telemedicine-oriented architecture. These characteristics extend the functionality of conventional wearable ECG systems while preserving compatibility with standard twelve-lead electrocardiographic assessment.

Several limitations of the present study should be acknowledged. First, the clinical evaluation was performed on a relatively limited cohort of 30 participants, which restricts the generalizability of the obtained findings. In addition, none of the participants had a known history of cardiovascular disease. Therefore, the present clinical comparison primarily evaluates ECG acquisition, signal quality, and measurement agreement with the reference system and should not be interpreted as a clinical validation of myocardial ischemia or infarction detection. Validation in patients with confirmed cardiovascular disease, including ischemic heart disease and myocardial infarction, is required in future studies. Second, the present study primarily demonstrates the technical feasibility and clinical applicability of the proposed platform rather than establishing diagnostic performance for specific cardiovascular diseases. In addition, the rule-based ST-segment and ischemia-classification algorithm was not benchmarked against an independent annotated multilead ECG database in the present study. Therefore, sensitivity, specificity, and positive predictive value for ischemic pattern detection cannot be established from the current results.

The ST_80_-based analysis presented here should consequently be interpreted as a functional evaluation of the implemented decision-support workflow rather than as evidence of diagnostic accuracy. External validation using established annotated multilead ECG databases, followed by prospective evaluation in patients with confirmed ischemic heart disease, is required in future work. A further limitation concerns direct quantitative validation of the lead-specific ST_80_ measurements. Although the BTL examination records contained lead-specific ST-level values, the measurement point used by the reference software could not be confirmed to correspond exactly to the J + 80 ms definition implemented in CardioService. These values were therefore not used for paired ST_80_ agreement analysis to avoid comparison of potentially non-equivalent measurements. Future validation should use a reference system or independently annotated ECG dataset in which ST measurements are defined at the same J + 80 ms point. Although representative cases of ST-segment abnormalities were successfully identified, larger multicenter studies involving diverse patient populations are required to determine diagnostic sensitivity, specificity, and overall clinical accuracy. An additional limitation concerns the conditions under which the clinical recordings were obtained. The present evaluation was performed under resting clinical conditions and did not include a dedicated ambulatory protocol involving walking or other controlled body movements. Consequently, the results reported here do not establish preservation of ECG signal quality during ambulatory use. Although the implemented preprocessing reduces baseline drift, power-line interference, and high-frequency noise, motion-related artifacts may overlap spectrally with the ECG signal and cannot be assumed to be completely removed by digital filtering. Evaluation of signal quality, electrode–skin stability, and the robustness of ECG feature extraction during controlled light ambulatory activities is therefore required in further validation of the wearable platform. Third, the current implementation depends on a PC-based host application for twelve-lead ECG reconstruction and automated signal analysis. The wearable unit independently performs synchronized acquisition, data buffering, local storage, display management, and wireless transmission; however, digital preprocessing, ECG reconstruction, interval measurement, ST_80_ assessment, contiguous-lead analysis, and preliminary report generation are currently performed on the host computer. Consequently, the present prototype should not be considered a fully standalone analytical system. This architecture was selected at the current research stage to facilitate modification and evaluation of signal-processing algorithms without redesigning the embedded firmware. Future development will focus on transferring selected reconstruction and analysis functions to the embedded platform or a mobile application to reduce dependence on an external computer. Finally, the current interpretation module is based predominantly on deterministic rule-based algorithms. Integration of advanced artificial intelligence and deep learning methods may further improve automatic detection of complex ECG abnormalities in future system versions.

An important limitation concerns the morphological comparison with the reference electrocardiograph. Recordings with the two systems were obtained sequentially rather than simultaneously, requiring repositioning of the electrodes between examinations. This is particularly relevant for the precordial leads, where differences in electrode position may influence QRS and T-wave morphology. Lead-specific quantitative analysis was consistent with the visual differences observed in Figure 10. Although R-wave amplitude showed relatively consistent agreement across most leads, agreement for T-wave amplitude was lower in V2–V4, with ICC values of 0.513, 0.446, and 0.475, respectively. Because simultaneous recordings using identical electrode positions were not available, the present study cannot determine whether these differences resulted primarily from variations in precordial electrode placement, device-related signal acquisition characteristics, or a combination of both. Therefore, the present data should not be interpreted as demonstrating morphological interchangeability between the two systems. Simultaneous acquisition using identical electrode positions will be required in future studies to establish waveform-level agreement more rigorously.

Overall, the proposed third-generation wearable ECG platform demonstrates that compact embedded hardware, synchronized twelve-lead ECG acquisition, automated multilead signal analysis, and real-time preliminary clinical interpretation can be successfully integrated into a single telemedicine-oriented system. The presented architecture represents an important step toward practical wearable electrocardiographic platforms capable of supporting continuous cardiovascular monitoring, early recognition of ischemic ECG changes, and efficient remote clinical decision support.

Second, the clinical validation focused primarily on the technical performance of the wearable platform, signal quality, waveform morphology, and agreement with a reference clinical electrocardiograph. Although the software successfully demonstrated automated ST-segment analysis and preliminary ECG interpretation, the present study was not designed to establish diagnostic sensitivity, specificity, or predictive performance for myocardial infarction or other cardiovascular diseases. Comprehensive diagnostic validation against expert cardiologist consensus and larger annotated clinical databases remains an important objective for future investigations.

Finally, the current software architecture is primarily based on deterministic signal-processing algorithms and rule-based ECG interpretation. Although this approach provides transparency, computational efficiency, and straightforward clinical implementation, incorporation of machine-learning and deep-learning techniques may further improve automatic recognition of complex ECG abnormalities and enhance diagnostic accuracy in future versions of the system.

## 5. Conclusions

This study presented the design, implementation, and preliminary clinical evaluation of a third-generation wearable twelve-lead electrocardiographic platform integrating synchronized multichannel ECG acquisition, automated signal processing, multilead ECG reconstruction, ST-segment analysis, and preliminary clinical interpretation within a unified telemedicine-oriented architecture. The proposed system combines a compact ESP32-S3-based embedded platform with dedicated software for ECG visualization, feature extraction, regional ST-segment assessment, and automated report generation.

The developed hardware architecture successfully reduced system complexity by integrating signal acquisition, wireless communication, local storage, and embedded control into a single microcontroller while maintaining reliable twelve-lead ECG acquisition. Clinical evaluation demonstrated successful acquisition of complete and interpretable twelve-lead ECG recordings in all participants, with good signal quality observed in the majority of recordings. Quantitative comparison with the reference electrocardiograph supported the feasibility of automated interval measurement; however, waveform-level morphological equivalence between the two systems was not established in the present study. These findings confirm the technical feasibility of the proposed wearable platform for multichannel ECG monitoring under routine clinical conditions.

The developed software framework provides automated ECG preprocessing, R-peak detection, feature extraction, twelve-lead reconstruction, ST_80_ measurement, regional analysis of contiguous ECG leads, and generation of a structured preliminary ECG report. Integration of these processing stages enables automatic assessment of electrocardiographic characteristics associated with myocardial ischemia while preserving physician oversight for the final clinical interpretation. The proposed workflow demonstrates the potential of combining wearable ECG acquisition with real-time computer-assisted analysis in a single integrated system.

Although the present study represents a pilot clinical evaluation, the obtained results demonstrate the feasibility of the proposed hardware and software architecture for wearable multilead electrocardiography. Future work will focus on validation in larger multicenter patient cohorts, quantitative assessment of diagnostic performance using expert-annotated clinical databases, and incorporation of advanced artificial intelligence methods to further improve automatic ECG interpretation and early detection of cardiovascular abnormalities.

Overall, the proposed platform represents a promising step toward next-generation wearable electrocardiographic systems capable of supporting continuous cardiac monitoring, telemedicine applications, and early clinical decision support for cardiovascular disease management.

## Figures and Tables

**Table 1:** Hardware architecture and technical characteristics of the three generations of the developed wearable ECG platform.

Parameter	First Generation (Sensors 2024)	Second Generation (Sensors 2026)	Proposed System
Primary application	ECG monitoring and arrhythmia detection	Multilead ECG analysis for glycemic status assessment	Spatial ECG analysis for ischemia-related ECG pattern assessment
Number of electrodes	4	10	10
Number of recorded ECG leads	1	12	12
Analog front-end	ADS1298	ADS1298	ADS1298
Main controller	STM32L151xD	STM32F103RC	ESP32-S3
CPU architecture	32-bit Arm Cortex-M3	32-bit Arm Cortex-M3	Dual-core 32-bit Xtensa LX7
Maximum CPU frequency	32 MHz	72 MHz	240 MHz
On-chip SRAM	48 KB	64 KB	512 KB
Program memory	384 KB Flash	256 KB Flash	384 KB ROM; SPI Flash capacity depends on the specific chip/module configuration
Wireless communication	GSM, Bluetooth	Wi-Fi (ESP32) + Bluetooth HC-05	Integrated Wi-Fi and Bluetooth Low Energy
Data storage	microSD	microSD	microSD
Embedded display	No	OLED display	OLED display
Signal processing architecture	Single-lead processing	Multilead processing	PC-based multilead reconstruction and spatial ECG analysis
USB connectivity	No	USB-C	Native USB OTG
PCB architecture	Distributed	Distributed	Integrated
Power consumption	—	—	~0.1 W (≈30 mA)
Diagnostic capability	Heart rate, HRV, arrhythmias	Multilead repolarization analysis	ST-segment analysis and ischemic pattern assessment

**Table 2:** Main software modules of the proposed wearable ECG platform.

Software Module	Main Functions
Embedded firmware	Initializes hardware peripherals, configures the ADS1298 analog front-end, acquires synchronized multichannel ECG signals, manages interrupts, and controls device operation.
Communication module	Transmits ECG data via integrated Wi-Fi and Bluetooth Low Energy, synchronizes data with the host application, and maintains reliable wireless communication.
Data buffering and storage	Buffers incoming ECG samples in memory and stores ECG recordings on the onboard microSD card to prevent data loss during communication interruptions.
Host application	Receives ECG recordings, reconstructs the standard twelve-lead ECG, manages examination records, and provides the user interface for clinical assessment.
Signal processing module	Performs ECG preprocessing, signal quality assessment, feature extraction, interval measurement, and twelve-lead ECG reconstruction.
Automatic analysis module	Detects rhythm abnormalities, evaluates ST-segment deviations, estimates clinically relevant ECG parameters, and generates preliminary diagnostic information.
Visualization module	Displays synchronized twelve-lead ECG signals, ECG parameters, waveform annotations, and analysis results in real time.
Report generation module	Generates examination reports, exports ECG recordings in standard formats, archives examination data, and supports subsequent clinical review.
Clinical decision-support module	Integrates analysis results, provides preliminary ECG interpretation, and assists physicians during clinical evaluation without replacing expert judgment.

**Table 3:** Signal quality classification criteria.

Signal Quality	Criterion
Good	(SNR > 8)
Medium	(4 < SNR ≤ 8)
Poor	(2 < SNR ≤ 4)
Unusable	(SNR ≤ 2)
No contact	(σ(s) < 1 μV)

**Table 4:** Digital filters used for ECG signal preprocessing.

Filter	Type	Parameters	Purpose
High-pass	Butterworth, 2nd order	Cutoff frequency = 0.5 Hz	Removal of baseline wander
Notch	IIR Notch	50 Hz, Q = 30	Suppression of power-line interference
Low-pass	Butterworth, 4th order	Cutoff frequency = 40 Hz	Removal of high-frequency noise and muscle artifacts

**Table 5:** Electrocardiographic criteria used for automated myocardial infarction detection.

Diagnostic Parameter	Criterion	Clinical Significance
ST_80_ elevation	≥0.10 mV (1 mm)	Suggestive of myocardial injury
ST_80_ depression	≤−0.10 mV (−1 mm)	Suggestive of myocardial ischemia
Borderline ST deviation	0.05–0.10 mV	Requires additional evaluation
Contiguous lead involvement	≥2 adjacent leads	Regional ischemic pattern
Pathological Q wave	Present	Possible myocardial necrosis
T-wave inversion	Present (excluding physiological leads)	Repolarization abnormality
STEMI pattern	ST elevation in ≥2 contiguous leads	Suspected acute STEMI

**Table 6:** Diagnostic features integrated into the automatic ECG interpretation module.

Diagnostic Component	Automatically Evaluated Parameters	Clinical Purpose
Signal quality assessment	Signal-to-noise ratio, electrode contact, baseline stability	Verify recording reliability
Rhythm analysis	HR, RR variability, bradycardia, tachycardia, irregular rhythm, suspected atrial fibrillation, pauses, extrasystoles	Rhythm assessment
ECG interval analysis	PR, QRS, QT, QTc	Cardiac conduction assessment
Waveform morphology	Pathological Q wave, T-wave inversion, R- and T-wave amplitudes	Morphological evaluation
ST-segment analysis	ST_80_ deviation, elevation, depression, borderline changes	Detection of myocardial ischemia
Regional analysis	Anterior, inferior, and lateral contiguous lead groups	Localization of ischemic changes
Automatic interpretation	Preliminary ECG report and suspected STEMI pattern	Clinical decision support

**Table 7:** Demographic, anthropometric, and lifestyle characteristics of the study participants (n = 30).

Characteristic	Value
Participants, n	30
Age, years	26.2 ± 9.9
Age range, years	18–56
Male, n (%)	25 (83.3%)
Female, n (%)	5 (16.7%)
Height, m	1.77 ± 0.09
Weight, kg	73.8 ± 13.0
BMI, kg/m^2^	23.8 ± 4.6
Known cardiovascular disease, n (%)	0 (0%)
Current smokers, n (%)	7 (23.3%)
Rare alcohol consumption, n (%)	8 (26.7%)
Physically active lifestyle, n (%)	10 (33.3%)
Moderate physical activity, n (%)	19 (63.3%)
Sedentary lifestyle, n (%)	1 (3.3%)

**Table 8:** Technical characteristics of the proposed third-generation wearable ECG platform.

Parameter	Result
Main controller	ESP32-S3
Analog front-end	ADS1298
Number of electrodes	10
Reconstructed ECG leads	12
Sampling frequency	500 Hz
PCB dimensions	60 × 80 mm
Previous PCB dimensions	60 × 100 mm
PCB area reduction	20%
Approximate current consumption	30 mA
Approximate power consumption	0.1 W
Wireless interfaces	Wi-Fi and BLE
Local storage	microSD
Clinical recordings completed	30/30

**Table 9:** Summary of the clinical evaluation and signal-quality assessment.

Evaluation Outcome	Result
Participants evaluated	30
Complete twelve-lead recordings	30/30 (100%)
Clinically interpretable recordings	30/30 (100%)
Recordings rated as good quality	≥90%
Recordings excluded because of incomplete lead formation	0
Significant interpretation-limiting artifacts	0 reported
Recording speed	25 mm/s
Display sensitivity	10 mm/mV

**Table 10:** Quantitative agreement between CardioService and the BTL Flexi 12 ECG for automated ECG interval measurements (n = 30).

Parameter	BTL, Mean ± SD (ms)	CardioService, Mean ± SD (ms)	MAE (ms)	Bias (ms)	RMSE (ms)	Pearson r	95% LoA (ms)
PR	159.10 ± 12.35	158.37 ± 14.05	7.00	−0.73	8.24	0.807	−17.10 to 15.63
QRS	96.27 ± 5.34	97.20 ± 5.33	2.53	+0.93	3.53	0.789	−5.86 to 7.72
QT	375.00 ± 16.88	374.27 ± 19.24	10.40	−0.73	12.61	0.756	−25.83 to 24.36
QTc	419.77 ± 14.89	416.47 ± 17.73	9.57	−3.30	10.53	0.820	−23.23 to 16.63

**Table 11:** Lead-specific agreement between CardioService and the BTL Flexi 12 ECG for R-wave and T-wave amplitudes across 30 paired recordings.

ECG Lead	R-Wave Pearson r	R-Wave ICC	T-Wave Pearson r	T-Wave ICC
I	0.991	0.991	0.975	0.974
II	0.992	0.991	0.975	0.975
III	0.919	0.915	0.923	0.916
aVR	0.952	0.950	0.902	0.904
aVL	0.971	0.965	0.935	0.895
aVF	0.992	0.991	0.980	0.979
V1	0.951	0.951	0.912	0.904
V2	0.953	0.926	0.805	0.513
V3	0.898	0.856	0.716	0.446
V4	0.857	0.772	0.727	0.475
V5	0.955	0.955	0.925	0.920
V6	0.988	0.988	0.959	0.958

**Table 12:** Comparison of the proposed wearable ECG platform and the reference clinical electrocardiograph.

Parameter	Proposed Wearable ECG Platform	Reference Clinical Electrocardiograph	Observation
Number of ECG leads	12 reconstructed leads	Standard 12 leads	Equivalent lead configuration
Number of electrodes	10	10	Identical electrode configuration
Sampling frequency	500 Hz	500 Hz	Comparable temporal resolution
ECG acquisition	Simultaneous multichannel acquisition	Simultaneous multichannel acquisition	Comparable acquisition principle
Signal quality	Good in >90% of recordings	Clinical reference	Diagnostic waveform quality achieved
P wave visualization	Clearly identifiable	Clearly identifiable	Visualized in both systems
QRS complex	Clearly identifiable	Clearly identifiable	Visualized in both systems; precordial morphology differed in some leads
T wave visualization	Clearly identifiable	Clearly identifiable	Visualized in both systems; direct morphological equivalence was not established
Twelve-lead reconstruction	Automatic	Native acquisition	Equivalent clinical presentation
ST-segment assessment	Automatic ST_80_ measurement with contiguous-lead analysis	Visual clinical assessment	Different implementation; same assessment objective
Rhythm analysis	Automatic	Physician interpretation	Decision-support function
Report generation	Automatic preliminary report	Manual clinical interpretation	Computer-assisted reporting

**Table 13:** Comparison of the proposed CardioService software with contemporary approaches for ECG interval assessment and validation.

Study/System	ECG Configuration	Validation Sample	Parameters Evaluated	Validation Approach	Main Quantitative Findings
De Bie et al. [21]	Standard 12-lead ECG; seven commercial interpretation programs	>2000 ECGs	PR, QRS, QT	Inter-program comparison against corrected program median	75th-percentile absolute errors: 4–6 ms (PR), 4–8 ms (QRS), 6–10 ms (QT)
van der Zande et al. [22]	Smartwatch-derived frontal/precordial ECG vs. standard 12-lead ECG	200 subjects	PR, QRS, QT; waveform amplitudes	Bland–Altman analysis: bias, absolute offset, 95% LoA	ECG interval durations obtained with the smartwatch were comparable with standard 12-lead ECG measurements
Ernstsson et al. [23]	Single-lead smartwatch ECG vs. standard 12-lead ECG	Pediatric cohort	PR, QRS, QT, QTc	ICC and Bland–Altman analysis	Automated measurements showed ICC = 0.86 (PR), 0.70 (QRS), 0.80 (QT), 0.53 (QTc)
Lu et al. [24]	Portable 12-lead ECG vs. conventional 12-lead ECG	62 patients	HR, PR, QRS, QT, QTc	Blinded clinical assessment and Bland–Altman analysis	HR, QRS, QT, and QTc measurements showed close agreement with conventional ECG; the PR difference was statistically significant but considered clinically small
CardioService (present study)	Reconstructed 12-lead ECG	30 patients	PR, QRS, QT, QTc; ST_80_ analysis	BTL Flexi 12 ECG; MAE, RMSE, Pearson correlation and Bland–Altman analysis	MAE = 7.00 ms (PR), 2.53 ms (QRS), 10.40 ms (QT), 9.57 ms (QTc); r = 0.756–0.820, all *p* < 0.001

**Table 14:** Lead-specific ST-segment results in the representative case.

Lead	ST, mV	ST, mm	Classification
I	−0.001	−0.01	Normal
II	0.007	0.07	Normal
III	0.013	0.13	Normal
aVR	−0.003	−0.03	Normal
aVL	−0.003	−0.03	Normal
aVF	0.010	0.10	Normal
V1	0.194	1.94	Elevation
V2	0.262	2.62	Elevation
V3	0.163	1.63	Elevation
V4	0.080	0.80	Borderline
V5	0.037	0.37	Normal
V6	0.012	0.12	Normal

**Table 15:** Comparison of the proposed platform with representative wearable and commercial portable ECG technologies.

Characteristic	Smartwatch ECG [7]	Wearable ECG Patch [8]	Wearable/Holter-Type ECG [18]	Commercial Portable 12-Lead ECG, Biocare iE10 [25]	Proposed Platform (This Study)
Primary application	Opportunistic rhythm and AF screening	Continuous ambulatory ECG monitoring	Long-term ambulatory arrhythmia monitoring	Resting 12-lead ECG acquisition and computerized interpretation	Wearable multilead ECG acquisition and spatial ECG decision support
Number of ECG leads	Typically 1 ECG lead	Single-channel ECG	3 recorded leads; six limb leads derived in the reported wearable system	12 standard leads	12 reconstructed leads
Simultaneous multilead acquisition	No	No	Yes	Yes	Yes
Main diagnostic/analytical focus	Rhythm and AF detection	Rhythm monitoring and AI-assisted arrhythmia detection	Arrhythmia detection and remote cardiac-event monitoring	Standard 12-lead measurements and computerized ECG interpretation	ECG interval analysis, ST-segment assessment and spatial pattern analysis
Automatic ECG interval measurement	Limited/device dependent	Not the principal reported function	HR and rhythm-related analysis	Yes: ventricular rate, PR, QRS, QT/QTc and electrical axes	Yes: RR, PR, QRS, QT and QTc
Lead-specific ST-segment assessment	Not intended for routine multilead ST assessment	Not reported	Limited by lead configuration	ST-segment analysis supported; detailed lead-specific ST measurement parameters are not specified in the publicly available product specifications	Automatic ST_80_ measurement for each reconstructed lead
Automated assessment of anatomically contiguous leads	No	No	Not reported	Not explicitly reported in publicly available specifications	Yes
Automated myocardial-territory localization	No	No	Not reported	Not explicitly reported in publicly available specifications	Yes; anterior, inferior and lateral lead groups
Real-time/remote data transmission	Yes, device dependent	Yes, cloud/mobile platform	Yes, real-time remote transmission	Yes; Wi-Fi, Bluetooth and network connectivity	Yes; integrated Wi-Fi/BLE
Automatic ECG interpretation	Primarily rhythm/AF classification	AI-assisted arrhythmia detection	Automatic cardiac-event detection	Yes; Biocare CardioPro ECG analysis program	Structured preliminary ECG decision-support interpretation
Report/data management	Application dependent	Cloud-based platform	Remote expert-supporting platform	ECG reports; ECG/XML/JPEG/DICOM/PDF formats; PC software support	Structured preliminary report, visualization, storage and export
Research-oriented algorithm extensibility	Proprietary/device dependent	Research platform	Research system	Proprietary commercial platform	Modular signal-processing architecture designed for further algorithm development
Main distinguishing feature	High accessibility for rhythm screening	Low-power continuous wearable monitoring	Long-term real-time telemonitoring	Mature commercial portable 12-lead ECG with computerized interpretation	Integration of compact 12-lead acquisition with automated ST_80_ analysis, contiguous-lead evaluation and myocardial-territory localization

## Data Availability

The data presented in this study are available from the corresponding author upon reasonable request. The datasets are not publicly available because they contain clinical electrocardiographic recordings and patient-related information collected during the clinical evaluation, and their sharing is subject to institutional and ethical restrictions.

## Supplementary Materials

The following supporting information can be downloaded at: https://www.mdpi.com/article/10.3390/s26175510/s1, Figure S1: Complete component-level electrical schematic of the proposed wearable twelve-lead ECG device; Table S1: Lead-specific Bland–Altman agreement between CardioService and the BTL Flexi 12 ECG for R-wave and T-wave amplitudes across 30 paired recordings.

## Abbreviations

The following abbreviations are used in this manuscript:

ADC	Analog-to-Digital Converter
BLE	Bluetooth Low Energy
BPM	Beats Per Minute
ECG	Electrocardiogram
ESP32-S3	Espressif ESP32-S3 Microcontroller
GUI	Graphical User Interface
HR	Heart Rate
HRV	Heart Rate Variability
IIR	Infinite Impulse Response
PCB	Printed Circuit Board
PR	PR Interval
QRS	QRS Complex Duration
QT	QT Interval
QTc	Corrected QT Interval
R-R	R-to-R Interval
SDNN	Standard Deviation of Normal-to-Normal Intervals
SPI	Serial Peripheral Interface
ST_80_	ST-Segment Amplitude Measured 80 ms after the J Point
TCP/IP	Transmission Control Protocol/Internet Protocol
USB	Universal Serial Bus
Wi-Fi	Wireless Fidelity
