# Supplementary material for: Development and Clinical Evaluation of a Wearable 12-Lead Electrocardiographic Platform with Automated ECG Analysis for Telemedicine Applications
Source: Sensors (Basel). 2026 Aug 30;26(17):5510. doi: 10.3390/s26175510 (PMC13567980; doi:10.3390/s26175510)
Supplement: Supplementary file 1 [file sensors-26-05510-s001.zip › SM Figure S1 — копия 2.pdf]

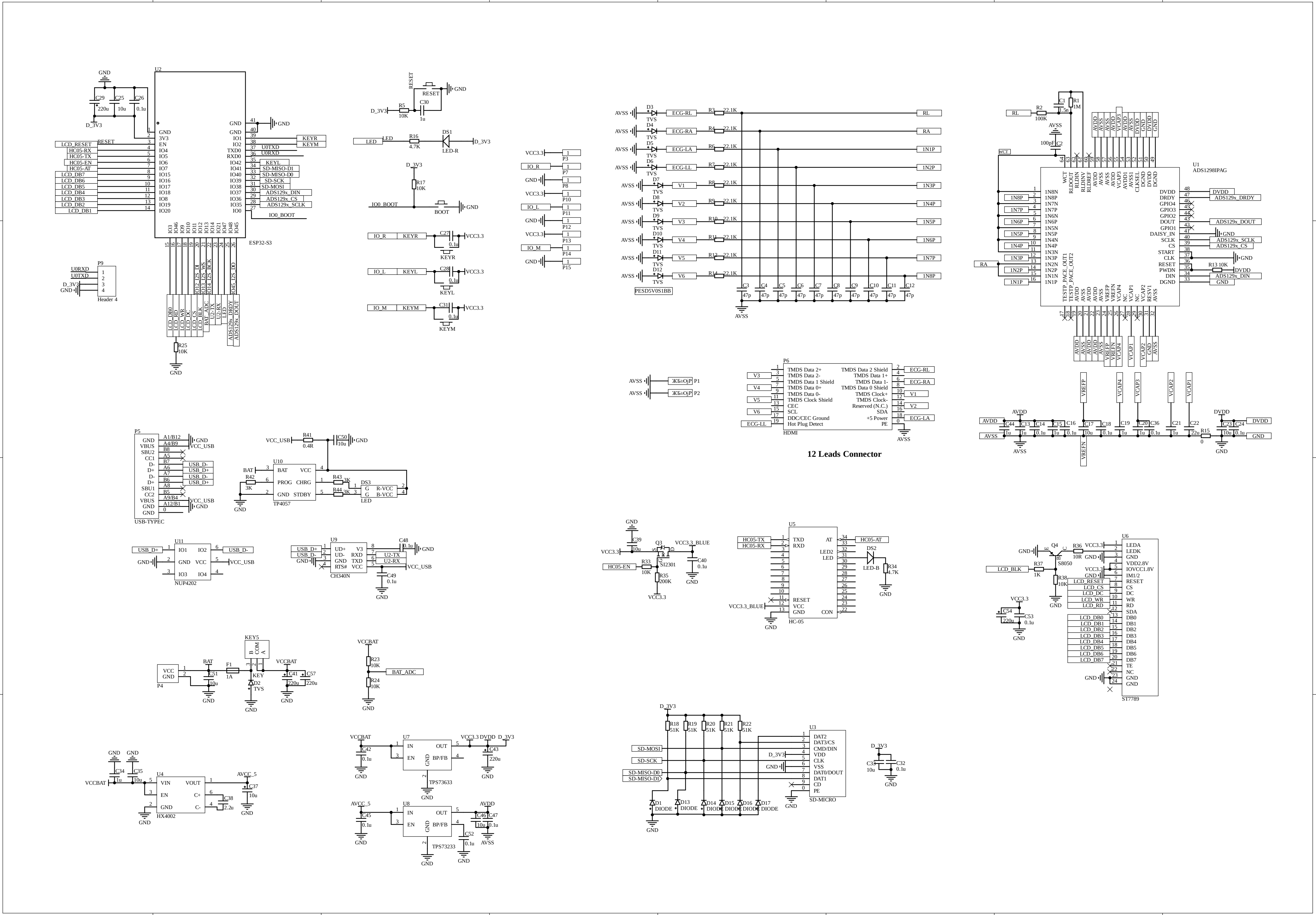
U2
ESP32-S3
GND
3V3
EN
IO4
IO5
IO6
IO7
IO15
IO16
IO17
IO18
IO8
IO19
IO20
IO3
IO46
IO9
IO10
IO11
IO12
IO13
IO14
IO21
IO47
IO48
IO45
IO1
IO2
TXD0
RXD0
IO42
IO41
IO40
IO39
IO38
IO37
IO36
IO35
IO0
RESET
LCD_RESET
HC05-RX
HC05-TX
HC05-EN
HC05-AT
LCD_DB7
LCD_DB6
LCD_DB5
LCD_DB4
LCD_DB3
LCD_DB2
LCD_DB1
KEYR
KEYM
U0TXD
U0RXD
KEYL
SD-MISO-D1
SD-MISO-D0
SD-SCK
SD-MOSI
ADS129x_DIN
ADS129x_CS
ADS129x_SCLK
IO0_BOOT
C29 220u
C25 10u
C26 0.1u
D_3V3
LCD_DB0
LCD_RD
LCD_WR
LCD_DC
LCD_CS
LCD_BLK
BAT_ADC
U2-TX
U2-RX
LED
ADS129x_DRDY
ADS129x_DOUT
R25 10K
P9
Header 4
RESET
C30 1u
R5 10K
R16 4.7K
DS1
LED-R
R17 10K
BOOT
IO_R
IO_L
IO_M
C27 0.1u
C28 0.1u
C31 0.1u
VCC3.3
P3
P7
P8
P10
P11
P12
P13
P14
P15
AVSS
D3
D4
D5
D6
D7
D8
D9
D10
D11
D12
TVS
ECG-RL
ECG-RA
ECG-LA
ECG-LL
V1
V2
V3
V4
V5
V6
R3
R4
R6
R7
R8
R9
R10
R11
R12
R14
22.1K
PESD5V0S1BB
RL
RA
1N1P
1N2P
1N3P
1N4P
1N5P
1N6P
1N7P
1N8P
C3
C4
C5
C6
C7
C8
C9
C10
C11
C12
47p
R2 100K
C1 1.5n
R1 1M
C2 100pF
WCT
U1
ADS1298IPAG
RLDOUT
RLDIN
RLDINV
RLDREF
AVDD
AVSS
VCAP3
AVDD1
AVSS1
CLKSEL
DGND
DVDD
DRDY
GPIO4
GPIO3
GPIO2
DOUT
GPIO1
DAISY_IN
SCLK
CS
START
CLK
PWDN
DIN
R13 10K
1N8N
1N8P
1N7N
1N7P
1N6N
1N6P
1N5N
1N5P
1N4N
1N4P
1N3N
1N3P
1N2N
1N2P
1N1N
1N1P
TESTP_PACE_OUT1
TESTN_PACE_OUT2
VREFP
VREFN
VCAP4
NC
VCAP1
VCAP2
RESV1
P1
P2
P6
TMDS Data 2+
TMDS Data 2-
TMDS Data 1 Shield
TMDS Data 0+
TMDS Data 0-
TMDS Clock Shield
CEC
SCL
DDC/CEC Ground
Hot Plug Detect
TMDS Data 2 Shield
TMDS Data 1+
TMDS Data 1-
TMDS Data 0 Shield
TMDS Clock+
TMDS Clock-
Reserved (N.C.)
SDA
+5 Power
PE
HDMI
12 Leads Connector
C44 1u
C13 1u
C14 0.1u
C15 1u
C16 0.1u
C17 10u
C18 0.1u
C19 1u
C20 1u
C36 0.1u
C21 1u
C22 22u
R15 0
C23 10u
C24 0.1u
P5
USB-TYPEC
GND
VBUS
SBU2
CC1
D-
D+
SBU1
CC2
A1/B12
A4/B9
B8
A5
B7
A6
A7
B6
A8
B5
A9/B4
A12/B1
USB_D-
USB_D+
VCC_USB
R41 0.4R
C50 10u
U10
TP4057
BAT
PROG
CHRG
STDBY
R42 3K
R43 3K
R44 3K
DS3
R-VCC
B-VCC
U11
NUP4202
U9
CH340N
UD+
UD-
RTS#
RXD
TXD
C48 0.1u
C49 0.1u
C39 10u
Q3
SI2301
VCC3.3_BLUE
C40 0.1u
R33 10K
R35 200K
U5
HC-05
AT
LED2
CON
DS2
LED-B
R34 4.7K
U6
ST7789
Q4
S8050
R36 10R
R37 1K
R38 10K
LEDA
LEDK
VDD2.8V
IOVCC1.8V
IM1/2
DC
WR
RD
TE
LCD_BLK
C54 220u
C53 0.1u
KEY5
COM
VCC
P4
F1 1A
C51 10u
D2 TVS
VCCBAT
C41 220u
C57 220u
R23 10K
R24 10K
U7
TPS73633
IN
OUT
BP/FB
C42 0.1u
C43 220u
DVDD
U8
TPS73233
AVCC_5
C45 0.1u
C46 10u
C47 0.1u
C52 0.1u
U4
HX4002
VIN
VOUT
C+
C-
C34 1u
C35 10u
C37 10u
C38 2.2u
U3
SD-MICRO
DAT2
DAT3/CS
CMD/DIN
VDD
CLK
VSS
DAT0/DOUT
DAT1
CD
R18 51K
R19 51K
R20 51K
R21 51K
R22 51K
D1
D13
D14
D15
D16
D17
DIODE
C33 10u
C32 0.1u
