# Supplementary material for: Development and Clinical Evaluation of a Wearable 12-Lead Electrocardiographic Platform with Automated ECG Analysis for Telemedicine Applications
Source: Sensors (Basel). 2026 Aug 30;26(17):5510. doi: 10.3390/s26175510 (PMC13567980; doi:10.3390/s26175510)
Supplement: Supplementary file 1 [file sensors-26-05510-s001.zip › SM Table S1.pdf]

Supplementary Materials

Table S1. Lead-specific Bland–Altman agreement between CardioService and the BTL Flexi 12 ECG for R-wave and T-wave amplitudes across 30 paired recordings.

ECG lead	R-wave bias (mV)	R-wave 95% LoA (mV)	T-wave bias (mV)	T-wave 95% LoA (mV)
I	+0.003	−0.039 to +0.045	+0.001	−0.017 to +0.020
II	−0.002	−0.045 to +0.040	+0.000	−0.019 to +0.019
III	+0.012	−0.081 to +0.106	+0.004	−0.022 to +0.031
aVR	+0.010	−0.071 to +0.091	−0.001	−0.030 to +0.028
aVL	+0.002	−0.066 to +0.070	+0.001	−0.035 to +0.037
aVF	+0.005	−0.037 to +0.046	+0.002	−0.013 to +0.018
V1	−0.006	−0.078 to +0.066	+0.003	−0.030 to +0.037
V2	−0.055	−0.192 to +0.081	+0.076	−0.032 to +0.185
V3	−0.078	−0.294 to +0.138	+0.080	−0.033 to +0.193
V4	−0.106	−0.354 to +0.143	+0.079	−0.042 to +0.201
V5	+0.006	−0.071 to +0.084	+0.004	−0.023 to +0.030
V6	+0.003	−0.045 to +0.051	−0.003	−0.024 to +0.019
